# Genetic Correlates of Presenile Dementia and Cognitive Decline in the Armenian Population Following COVID-19: A Case-Control Study

**DOI:** 10.3390/ijms26146965

**Published:** 2025-07-20

**Authors:** Yekaterina Hovhannisyan, Hermine Yeritsyan, Gohar Hakobjanyan, Gayane Petrosyan, Hayk Harutyunyan, Armen Muradyan, Allen Azizian, Konstantin Yenkoyan

**Affiliations:** 1 Neuroscience Laboratory, COBRAIN Center, Yerevan State Medical University Named after Mkhitar Heratsi, Yerevan 0025, Armenia; katyaarmdoc@yahoo.com (Y.H.); yeritsyan_hermine@yahoo.com (H.Y.); hayk@web.am (H.H.); 2 Neurology Service at Neurosurgery Comprehensive Stroke Center, Heratsi N1 University Clinic, Yerevan State Medical University Named after Mkhitar Heratsi, Yerevan 0025, Armenia; 3 Laboratory-Diagnostic Center Heratsi N1 University Clinic, Yerevan State Medical University Named after Mkhitar Heratsi, Yerevan 0025, Armenia; hakobjanyangm@gmail.com (G.H.); drgaiane.petrosyan@gmail.com (G.P.); 4 COBRAIN Center, Yerevan State Medical University Named after Mkhitar Heratsi, Yerevan 0025, Armenia; muradyan_a@yahoo.com; 5 Department of Urology and Andrology, Yerevan State Medical University Named after Mkhitar Heratsi, Yerevan 0025, Armenia; 6 Department of Criminology, California State University, Fresno, CA 93740, USA; aazizian@mail.fresnostate.edu; 7 Department of Medical Psychology, Yerevan State Medical University Named after Mkhitar Heratsi, Yerevan 0025, Armenia

**Keywords:** dementia, COVID-19, cognition, depression, gene, environment

## Abstract

The presence of cognitive lapses in the post-COVID-19 period, particularly among younger individuals, suggests a potential genetic predisposition. This case–control study aimed to assess the association between neurodegeneration-associated genes and cognitive declines in the post-COVID-19 Armenian population under the age of 65. In addition, we examined other contributing factors, including depressive symptoms, hypovitaminosis D, vitamin B12 and B9 deficiencies, and some viral infections, as potential confounders or effect modifiers. A total of 162 participants (ages 19–65, Med = 43), who were exposed to SARS-CoV-2 in Armenia between 2020 and 2022, participated in this study. Standardized assessments, including the Repeatable Battery for the Assessment of Neuropsychological Status (RBANS) and the Montreal Cognitive Assessment (MoCA), were used to evaluate cognitive functions and mental status, while the Patient Health Questionnaire-9 (PHQ-9) was utilized to assess depressive symptoms. Clinical interview data, comprising yes/no self-reports regarding the presence of cognitive problems and depressive symptoms, were also included. Genetic analysis identified copy number variations (CNVs) in the *APP*, *PSEN1*, *PSEN2*, *MAPT*, and *GRN* genes, while viral infections (HSV-1, HSV-2, CMV, EBV, HIV, SARS-CoV-2, Hepatitis A, B, and C) and vitamin D, B12, and B9 deficiencies were measured. Lower cognitive performance was associated with CNVs in *PSEN1* (exons 1, 9, 12), *GRN* (exons 1, 6, 12), and *MAPT* (exons 2, 8), along with viral infections (HSV-1, HSV-2, HAV-2). The findings indicate that post-COVID-19 cognitive problems are multifactorial and are linked to genetic mutations, viral infections, age, gender, and folic acid deficiency.

## 1. Introduction

Cognitive lapses, colloquially known as brain fog, impact global health, including the younger population [1]. It negatively affects quality of life, daily functionality, and has socio-economic consequences. Alzheimer’s disease (AD), the most common cause of dementia [2], typically presents in older adults, but early-onset AD (EOAD)—occurring before age 65—represents a rarer form (~2% of AD cases), primarily affecting younger individuals. This led us to investigate the main genetic mutations associated with EOAD, specifically those in the *Amyloid Precursor Protein* (*APP*), *Presenilin 1* (*PSEN1*), and *Presenilin 2* (*PSEN2*) genes [3]. *PSEN1* and *PSEN2* are integral to the γ-secretase complex, which processes APP and leads to the production of amyloid-beta (Aβ) peptides, a key factor in neuronal damage in AD [4]. Additionally, we explored two of the three main genetic mutations associated with frontotemporal dementia (*FTD*)—those in the Microtubule-Associated Protein tau (*MAPT*) and Progranulin (*GRN*) genes—as FTD is a neurodegenerative condition that often affects younger populations [5].

Thus, in light of the significant impact of cognitive lapses on younger populations, a primary goal of ours was to investigate the genetic predisposition to cognitive decline in individuals under the age of 65, with a particular focus on genetic copy number variations (CNVs). Given that age-related risk factors were not a concern in our selected younger cohort, we aimed to isolate the genetic components contributing to early-onset neurodegenerative diseases. To evaluate cognitive functioning, we employed both the Montreal Cognitive Assessment (MoCA) and the Repeatable Battery for the Assessment of Neuropsychological Status (RBANS), prioritizing MoCA due to its greater cultural adaptability and routine clinical use, despite its more limited domain specificity compared to RBANS.

While SNPs have been the predominant focus in post-COVID and neurodegenerative genetic studies, CNVs represent a distinct class of structural genomic variations with potentially greater functional impact due to dosage changes. Recent large-scale analyses have underscored this: a Genome Medicine study using UK and Estonian Biobank data found that rare CNVs significantly contribute to disease risk—often showing stronger effects on clinical phenotypes compared to SNPs [6]. A comprehensive review in *Human Genomics* further confirmed that rare CNVs affecting dosage-sensitive genes (e.g., *PSEN1*, *MAPT*, *GRN*) exhibit more pronounced impacts on gene expression and cognitive traits than single-nucleotide variants [7]. Importantly, ancestry-dependent differences in CNV burden have been reported, reinforcing the need for population-specific genomic investigations [8]. However, CNVs have been minimally explored in the context of post-COVID cognitive impairment—highlighting both the novelty and necessity of our CNV-focused approach to understanding individual susceptibility to cognitive decline following SARS-CoV-2 infection.

Specifically, we sought to explore the role of CNVs as potential biomarkers in the preclinical stages of conditions like AD, as previous studies have suggested their utility in identifying at-risk individuals before the onset of symptomatic cognitive decline [9]. CNVs are DNA segments of varying lengths, ranging from 1 Kb to several Mb, that can have different numbers of copies. These CNVs, which involve deletions or duplications of genomic segments, play a significant role in the development of complex conditions, including mental disorders [10,11], cognitive decline [12], and depressive symptoms [13] in adults.

In parallel with genetic risk markers, recent research underscores the importance of neurophysiological mechanisms—particularly cortical excitability—as early indicators of cognitive vulnerability. Techniques such as transcranial magnetic stimulation (TMS) have demonstrated that altered excitatory/inhibitory dynamics in cortical circuits, especially in the prefrontal cortex, can reflect subclinical cognitive disturbances in aging and neurodegenerative disease [14,15]. These functional biomarkers may offer complementary insights into genomic data, supporting a more integrated understanding of resilience and decline in cognitive performance

In our study, we directly analyzed CNVs across 14 exons of the MAPT gene, motivated by its well-established role in tauopathies and early-onset neurodegeneration. The MAPT gene was used to assess its relationship with cognitive function, given its well-established role in tauopathies and early-onset neurodegeneration.

The *MAPT* gene encodes the tau protein, which has important roles in regulating axonal transport, synaptic activity, cytoskeletal dynamics, and cell signaling [16]. In humans, the tau protein undergoes alternative splicing to generate six isoforms with distinct functions [17]. *MAPT* gene exons 1, 4, 5, 7, 9, 11, and 13 are constitutive exons, meaning they are expressed in all six isoforms of the tau protein. Exon 1 encodes a portion of tau’s N-terminal region, which plays a critical role in axonal transport and facilitates tau’s interaction with the cytoplasmic membrane and signaling proteins [18]. Exons 4, 5, 7, and part of exon 9 encode the region between the N-terminal inserts and the first microtubule-binding repeat. This region contains proline-rich segments predominantly encoded by exons 7 and 9. The three constitutive tubulin-binding repeats are encoded by exons 9, 11, and 12, while exon 13 contributes to the C-terminal region of tau [19,20]. Conversely, exons 2, 3, 4A, 6, 8, and 10 undergo alternative splicing. As a result, six major tau isoforms are found in the human central nervous system (CNS) due to different splicing combinations of exons 2, 3, and 10 [17]. Notably, exon 8 has not been shown to be transcribed in human mature MAPT mRNA [18,21], and its exclusion is considered the default splicing pattern, as demonstrated by exon-trapping experiments [22]. Pathogenic mutations in the MAPT gene are primarily located within exons 9 to 13. In fact, even silent and intronic mutations can contribute to the development of tauopathies [23].

While pathogenic *MAPT* mutations are well-characterized within exons 9–13, and most *GRN* CNVs are known to cause haploinsufficiency in frontotemporal dementia, the specific roles of CNVs in *MAPT* exon 8 and *GRN* exon 6 remain poorly defined. For example, *GRN* exon-level deletions—including exon 6—have been observed in FTD families and are considered pathogenic through transcript loss and reduced Progranulin expression [24,25,26]. By contrast, exon 8 of *MAPT* is typically excluded from mature transcripts, and no functional consequences of its CNVs have been documented [27]. These gaps highlight the need for the functional validation and cautious interpretation of exon-level CNVs, especially under conditions of viral or neuroinflammatory stress.

The signaling pathways involved in cell growth, proliferation, metabolism, cell-to-cell interactions, regeneration, apoptosis, and stress responses play critical roles in cellular function. Under stress conditions, interactions between these pathways and tau proteins can lead to either adaptive responses or apoptosis, with outcomes that may be neuroprotective or neurodegenerative [16]. In this context, the study by Ruiz-Gabarre et al. identified the N-terminal region of tau proteins as the primary binding site for these interactions [18]. This region, which is partially encoded by exon 1 of the *MAPT* gene (non-alternatively spliced), suggests that mutations in exon 1 (leading to a partially deficient N-terminal region) could impair interactions with signaling systems, potentially contributing to neurodegeneration under stress. One example of such stress is exposure to SARS-CoV-2.

According to the study by Violet et al. [28], tau plays a protective role in maintaining neuronal DNA and RNA integrity during oxidative stress. Alterations in tau can lead to neuronal damage under such stress, which may be a critical factor in the pathogenesis of SARS-CoV-2 infection, as suggested by Wieczfinska et al. [29]. Additionally, the research by Didonna et al. highlights tau’s protective role during neuroinflammation [10]. These findings may help explain why individuals with *MAPT* gene mutations could be more vulnerable to neuronal damage and subsequent cognitive impairment following exposure to SARS-CoV-2.

Current research on *MAPT* mutations primarily focuses on small nucleotide polymorphisms (SNPs) that lead to tauopathies and synucleinopathies [30,31]. However, there are a lack of comprehensive data regarding the role of exonic CNV mutations in gene regulation [21].

Our study coincided with the onset of the COVID-19 pandemic [32], during which SARS-CoV-2 emerged as a potential risk factor for cognitive impairment. In the acute phase, 52.31% of patients with COVID-19 experienced cognitive deficits [13]. These deficits manifested in a range of symptoms that significantly impacted daily functioning, including brain fog (32%), memory decline (17.5–35%), and attention impairment (22%) [33]. Post-COVID-19 cognitive impairment has been reported with prevalence rates varying from 7.2% to 59.2%. However, the underlying predisposing factors, mechanisms, and effective treatment approaches remain unclear [34]. In addition, we considered other risk factors contributing to cognitive impairment, including depression [35,36], hypovitaminosis D [37], and deficiencies in vitamin B12 [38] and B9 [39]. We also examined the potential role of human herpesvirus infections, such as herpes simplex virus type 1 and 2 (HSV-1 and HSV-2), cytomegalovirus (CMV), Epstein–Barr virus (EBV) [40], as well as Hepatitis A [41], Hepatitis B [42], Hepatitis C [43], and HIV [44]. These factors were tested as potential confounders and/or effect modifiers.

Thus, this case–control study aimed to evaluate the contribution of neurodegeneration-associated gene copy number variations to cognitive decline in the Armenian Population Following COVID-19 aged 19 to 65, while also examining the role of depressive symptoms, hypovitaminosis D, vitamin B12 and B9 deficiencies, and viral infections as potential confounders or effect modifiers.

## 2. Results

A total of 162 patients were included in this study, with 35% male and 65% female participants.

### 2.1. Virological Results and Hypovitaminosis

The following positive virological results and hypovitaminosis statuses were observed in the study population: CMV IgG (97%), HIV (0.7%), HSV-1 (67%), HSV-2 (7%), A-HCV (0.7%), EBV IgG (90%), HBSAG (3.4%), A-HAV 2 (83%), A-COV2 IgG (96%). The prevalence of hypovitaminosis was as follows: B12 (14%), D (48%), B9 (25%) (detailed information available in Supplementary Table S1).

### 2.2. Genetic Testing Results

Genetic testing was performed on 13 exons of *MAPT*, 5 exons of *GRN*, 18 exons of *APP*, 12 exons of *PSEN1*, and 13 exons of *PSEN2* genes. The results showed the following distributions of homozygous CNV mutations: *MAPT* exon 1 (62%), exon 2 (2.6%), exon 6 (4.6%), exon 8 (1.3%), exon 12 (0.7%), 13 (0.7%), 14 (0.7%), *GRN* exon 6 (4.6%), exon 10 (7.9%), exon 12 (7.9%), *APP* exon 1 (64%), *PSEN1* exon 1 (11%), exon 6 (1.3%), exon 12 (1.3%), *PSEN2* exon 1 (84%), exon 2 (26%), exon 9 (0.7%), exon 11 (1.3%).

In addition, heterozygous CNV mutations were more common, with notable mutations in the following exons: *MAPT* exon 1 (26%), exon 3 (0.7%), exon 4 (0.7%), exon 5 (1.3%), exon 6 (89%), exon 7 (2.6%), exon 8 (15%), exon 9 (11%), exon 10 (42%), exon 11 (25%), exon 12 (95%), 13 (12%), 14 (20%); *GRN* exon 3 (11%), exon 6 (72%), exon 10 (85%), exon 12 (79%); *APP* exon 1 (32%), exon 3 (2.6%), exon 4 (21%), exon 5 (1.3%), exon 6 (2.0%), exon 7 (2.0%), exon 8 (4.6%), exon 12 (0.7%), exon 13 (1.3%), exon 14 (3.3%), exon 15 (12%), exon 16 (8.6%), exon 17 (43%), exon 18 (0.7%); *PSEN1* exon 1 (51%), exon 2 (8.6%), exon 3 (0.7%), exon 5 (0.7%), exon 6 (6.6%), exon 7 (4.0%), exon 8 (2.0%), exon 9 (0.7%), exon 10 (0.7%), exon 12 (11%); *PSEN2* exon 1 (16%), exon 2 (63%), exon 3 (11%), exon 4 (5.3%), exon 5 (13%), exon 6 (50%), exon 7 (16%), exon 8 (15%), exon 9 (56%), exon 10 (40%), exon 11 (68%), exon 12 (67%), exon 13 (60%).

No CNV mutations were detected in the following exons: *APP* exon 2, 9, 10, 11; *PSEN1* exon 4 and 11 (Supplementary Table S1).

### 2.3. Cognitive Status and CNV Mutations

**Figure 1:**
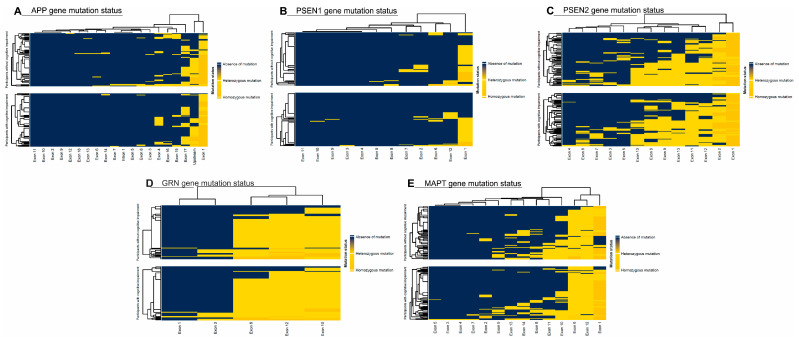
(**A**) *Amyloid Precursor Protein* (*APP*), (**B**) *Presenilin 1* (*PSEN1*), (**C**) *Presenilin 2* (*PSEN2*), (**D**) *Progranulin* (*GRN*), and (**E**) *Microtubule-Associated Protein tau* (*MAPT*) gene distribution.

**Figure 2:**
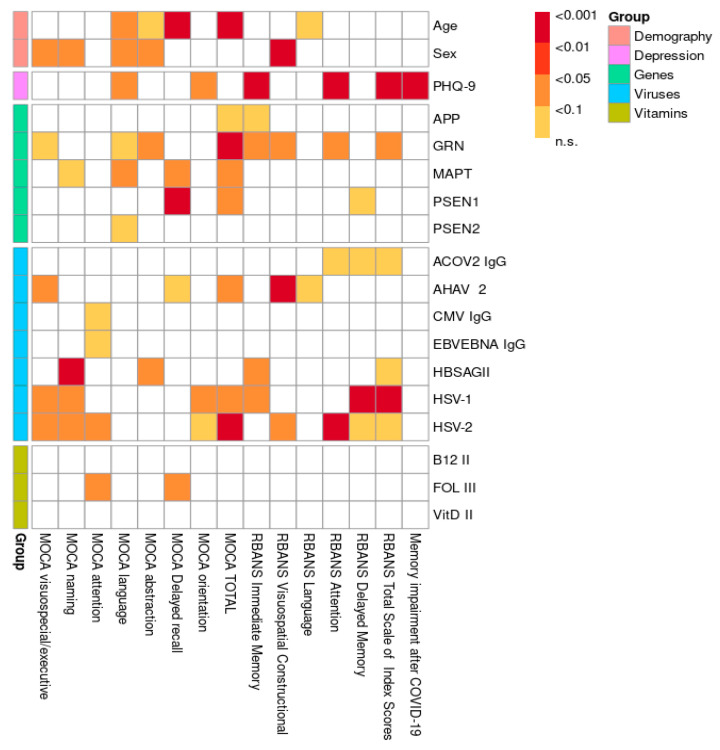
Visualization of *p*-values without multiple comparison adjustment.

**Figure 3:**
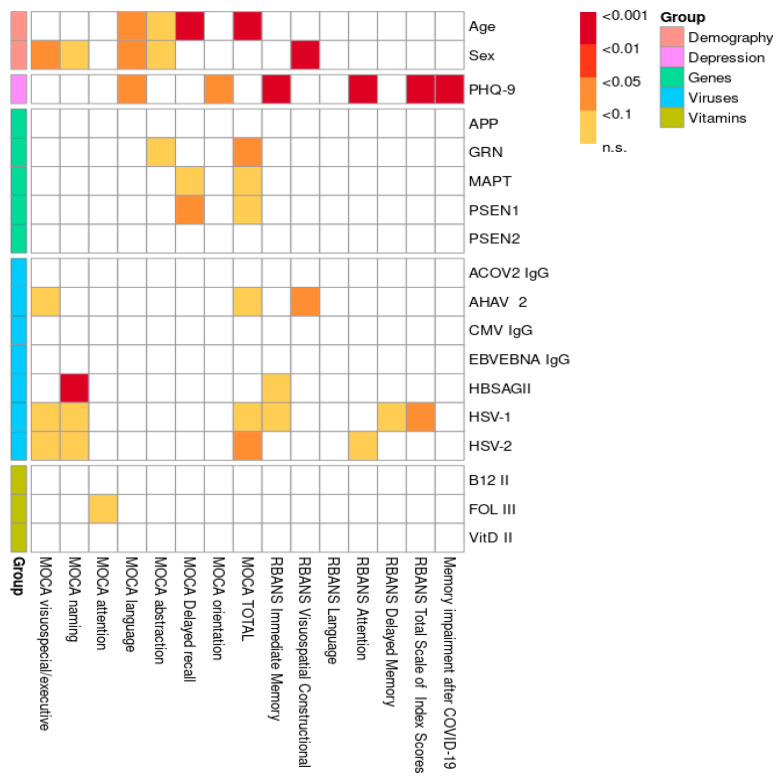
Visualization of *p*-values with multiple comparison adjustment.

The distribution of CNV mutations in the *APP*, *PSEN1*, *PSEN2*, *GRN*, and *MAPT* genes, stratified by cognitive status as measured by the MoCA test, is shown in Figure 1. A comparison of the included risk factors (age, gender, genetic mutations, viruses, hypovitaminosis) was conducted between total neurocognitive test scores (MoCA and RBANS) and across different cognitive domains, as shown in Figure 2. The results were adjusted for multiple comparisons, revealing a significant change in the findings, as shown in Figure 3.

The overall statistical comparison revealed a strong association between the total MoCA score, short-term memory, and age, which remained significant after adjustment (*p* < 0.001). Similarly, both the MoCA and RBANS results showed a significant association between age and speech impairment in the study population, which persisted after adjustment (*p* < 0.01). A weak association was observed between abstract thinking and age according to the MoCA results (*p* < 0.1), even after adjustment. Furthermore, both the MoCA (*p* < 0.05) and RBANS (*p* < 0.001) results indicated that men had lower scores and exhibited greater overall cognitive impairment compared to women. After adjustment for multiple comparisons, a statistically significant relationship was observed between speech dysfunction and men (*p* < 0.05), along with a weak association with abstract thinking and naming (*p* < 0.1). Our research demonstrates a significant correlation between depression and cognitive impairment in young and middle-aged individuals, as evidenced by the RBANS results both before and after adjustment. Specifically, memory (*p* < 0.001) and attention (*p* < 0.001) were particularly impaired in the context of depression. Furthermore, complaints of cognitive impairment following COVID-19 were significantly associated with a depressive state (*p* < 0.001). Several associations between CNV mutations in genes (*APP*, *GRN*, *MAPT*, *PSEN1*, *PSEN2*) and various domains of cognitive impairment were identified. A few remained significant after adjustment for multiple comparisons: *GRN* gene mutations were associated with abstract thinking (*p* < 0.1), while *MAPT* and *PSEN1* mutations were linked to memory impairment (*p* < 0.1 and *p* < 0.05, respectively), as shown in Figure 3 and Supplementary Table S2. Additionally, all three genes were significantly associated with the MoCA total score. Interestingly, based on our research data, we suggest that the MoCA may be a more sensitive test for assessing cognitive impairment in the studied population compared to the RBANS. A detailed statistical analysis of the different exons of the *PSEN1* (Table 1A,B), *MAPT* (Table 2A,B), and *GRN* (Table 3A,B) genes in relation to the significantly associated cognitive impairment domains was conducted based on MoCA data. For the *PSEN1* gene, a model–submodel test showed a significant association of MoCA delayed recall with exon 1 (*p* = 0.0070) with an effect size compatible with 95% CI [−2.4, −0.48]. For the MoCA total score, associations were found for exon 9 and exon 12 (*p* = 0.026 and *p* = 0.027 according to model–submodel tests correspondingly). For exon 9, a 95% CI of [0.98, 15.00] was detected for a homozygous type. Unfortunately for a contrast test, exon 12 did not reach significance (CI [−9.90, 0.12]). This discrepancy may reflect limited statistical power or a true lack of effect; accordingly, definitive conclusions should await validation in larger cohorts. According to the model–submodel test, CNV mutations in *GRN* exon 1 (*p* = 0.027) and exon 6 (*p* = 0.0029) may be associated with a specific cognitive impairment expressed by a MoCA abstract score. However, an analysis of contrasts shows that the potential effect size is rather modest (95% CI is [0.045, 0.73] for exon 1 and [0.160, 0.810] for exon 2. For the MoCA total score, the model–submodel test highlights associations with exon 6 (*p* = 0.00015) and exon 12 (*p* = 0.00130). The test of contrasts shows that a single mutation in exon 6 is associated with a change in the MoCA total score ranging from 0.95 to 6.90, while a mutation in exon 12 is associated with a change ranging from −6.20 to −0.97. Regarding the impact of CNV mutations in the *MAPT* gene on cognitive impairment, a statistically significant association with the MoCA delay recall was found for exon 2 (*p* = 0.014) and with the MoCA total score for exon 8 (*p* = 0.033). The contrast analysis of effect sizes results in a 95% CI of [0.49, 4.8] and [−13, −1], correspondingly. With regard to the impact of hypovitaminosis (B9, B12, D) and viruses on objective cognitive decline (MoCA; RBANS), after adjustment, associations were found between HSV-1 (*p* < 0.05), HSV-2 (*p* < 0.05), and AHAV-2 (*p* < 0.01). Infections were identified as possible confounders for cognitive impairment in different domains: Hepatitis B for naming (*p* < 0.001) and memory (*p* < 0.1); Hepatitis A for executive function (*p* < 0.05); HSV-1 for executive function, naming, and memory (*p* < 0.1); and HSV-2 for executive function, naming, and attention (*p* < 0.1). A weak association was observed between folate (vitamin B9) deficiency and attention (*p* < 0.1). Hypovitaminosis B12 and D, however, did not show an effect on cognitive dysfunction in our study population (Figure 3; Supplementary Table S2).

## 3. Discussion

This study examined the association between neurodegeneration-related genetic markers and post-COVID-19 cognitive impairments. The sample consisted of a cohort of individuals of Armenian ethnicity who were exposed to SARS-CoV-2 in Armenia between 2020 and 2022. Notably, this cohort was demographically distinct, representing a younger age group (Med = 43) than typically seen in studies of neurodegeneration-related genetic markers. We investigated mutations linked to early-onset Alzheimer’s disease (EOAD), particularly in the *APP*, *PSEN1*, and *PSEN2* genes, which affect amyloid-beta production and neuronal integrity. Additionally, mutations in the *MAPT* and *GRN* genes—commonly associated with frontotemporal dementia (FTD), a condition that disproportionately affects younger individuals—were analyzed. Finally, this study compared self-reported cognitive lapses with standardized assessments to evaluate how these measures correlate and whether their relationship is influenced by clinical and demographic variables.

AD is characterized by the accumulation of amyloid-β (Aβ) in plaques and abnormally phosphorylated tau in neurofibrillary tangles [12]. Aβ accumulation begins up to two decades before the onset of dementia [45]. EOAD is caused by mutations in three key genes: *APP*, *PSEN1*, and *PSEN2* [46]. According to ACMG-AMP guidelines, *PSEN1* mutations are the most common cause of AD in patients (89.16%), followed by APP mutations (46.97%).

The *APP* gene, located on chromosome 21q, contains 18 exons and encodes a protein involved in synaptic activity, transcriptional regulation, plasticity, and neuroprotection [47]. Genetic alterations in *APP* increase protein levels, leading to early-onset AD. Exons 7, 8, and 15 are subject to alternative splicing [48], and duplications or triplications of the *APP* gene can result in familial Alzheimer’s disease (fAD) [49,50]. Mutations in *APP* are most prevalent in exons 14, 16, and 17, and no variants are found in exons 1, 2, 3, 4, 8, 10, 15, and 18, which aligns with previous studies [51]. Mutations in exon 16 affect γ-secretase cleavage regions, influencing Aβ42 and Aβ40 ratios crucial for amyloid plaque formation [52]. As the catalytic component of the γ-secretase complex is encoded by *PSEN1* and *PSEN2*, mutations in those genes disrupt APP processing, leading to increased Aβ accumulation [53]. It is also interesting that abnormal axodendritic proliferation has been observed in neurons with a *PSEN1* mutation, which is explained by elevated levels of the cytoplasmic APP C-terminal fragments [54].

### 3.1. Cognitive Functioning and Depressive Symptoms

One of the broader aims of our study was to examine whether genetic factors could help explain why some COVID-19 patients experience cognitive lapses while others do not. Our findings showed that *PSEN1* CNV mutations in exons 1, 9, and 12 were associated with specific cognitive deficits and helped distinguish patients based on the presence and type of cognitive impairment. The most prominent finding was mild indicators of memory problems, which are commonly reported by individuals who have had COVID-19. Other cognitive domains also showed statistically significant associations; however, our team did not assign them strong weight, given the multiple comparisons. Further specification and standardization of memory assessments is warranted, given the lack of consistency in our findings between MoCA and RBANS—partly due to different tests measure memory using varying methods and criteria.

Our findings suggest that depressive symptoms contributed to post-COVID-19 cognitive decline, as reflected in both performance-based impairments in memory and individuals’ self-reported difficulties with thinking and mental clarity. This raises questions about the origin of cognitive problems in COVID-19: Is it primarily a consequence of the psychomotor slowing commonly associated with depression, which then impacts cognitive functioning, or does it reflect a direct cognitive deficit independent of mood symptoms? We hypothesize that for the general population, the former is more likely—particularly in the context of post-COVID-19 ‘cognitive fog,’ which may reflect slowed processing and attention linked to depressive symptomatology rather than isolated neurocognitive damage. However, for individuals with a genetic predisposition to neurodegeneration, COVID-19 may act as a catalyst, accelerating underlying pathological processes and resulting in more pronounced and persistent cognitive impairment.

To summarize, we identified CNV-associated cognitive impairments involving *PSEN1* (exons 1, 9, and presumably 12), *GRN* (exons 1, 6, 12), and *MAPT* (exons 2 and 8), which is consistent with prior findings by Xiao et al. [29] reporting pathogenic *PSEN1* variants in exons 4–8, 11, and 12.

We hypothesize that *PSEN1* exon 12 CNV mutations, common in our cohort, could potentially be pathogenic for the Armenian population. However, as noted in the Results Section, the inconsistency between the model–submodel test and the contrast test for exon 12 underscores the need to investigate this phenomenon in larger cohorts. Given the limited statistical power and wide confidence intervals, the observed association should be interpreted with caution and considered hypothesis-generating until validated in independent samples. Most of the *PSEN2* variants were found in exon 5, exon 7, and exon 4, and no variants were detected in exon 1, exon 2, and exon 3 [51]. However, in the current study, no significant associations were found with CNV mutations in *APP*, *PSEN1*, or *PSEN2* genes and cognitive decline outside of these exons. Notably, we did not detect CNV mutations in *APP* exons 2, 9, 10, or 11, nor in *PSEN1* exons 4 and 11, which is in line with previous reports [51].

Another important cause of early-onset dementia is frontotemporal lobar degeneration (FTLD), which is often associated with familial forms of the disease [5,55]. It is a highly heritable autosomal dominant disorder [56] and presents with different clinical forms: behavioral variant FTD (bvFTD), the semantic variant (svPPA), and the nonfluent variant (nfPPA) of primary progressive aphasia [11]. The *GRN* and *MAPT* genes are key contributors to FTLD [5,55]. In a Belgian cohort of familial FTD patients, *PGRN* mutations were found to be 3.5 times more prevalent than *MAPT* mutations, underscoring the significant role of PGRN in the etiology of FTD [57]. In contrast, the *GRN* and *MAPT* mutations are relatively uncommon in Russia [58].

In our study of the Armenian population, we identified both homozygous and heterozygous CNV mutations in the *MAPT* gene across several exons. Homozygous mutations were found in exon 1 (62%), exon 2 (2.6%), exon 6 (4.6%), exon 8 (1.3%), exon 12 (0.7%), exon 13 (0.7%), and exon 14 (0.7%). Heterozygous mutations were observed in exon 1 (26%), exon 3 (0.7%), exon 4 (0.7%), exon 5 (1.3%), exon 6 (89%), exon 7 (2.6%), exon 8 (15%), exon 9 (11%), exon 10 (42%), exon 11 (25%), exon 12 (95%), exon 13 (12%), and exon 14 (20%). Despite the fact that exon 8 has not been identified in cortical tau from humans [59], our study observed a statistical association between CNV mutations in exon 8 of the *MAPT* gene and cognitive decline. However, given the exploratory nature of this analysis and the relatively small number of individuals carrying these mutations, this association should be interpreted cautiously. According to the Ensembl database, transcripts containing exon 8 encompass all alternative exons except exon 0, which may either be included or excluded [21]. This raises a compelling question as to how CNV mutations in exon 8 could contribute to cognitive decline. While exon 8 is generally excluded in mature *MAPT* transcripts in the adult human cortex [48,60], its presence has been documented in certain alternative isoforms expressed under non-physiological or stress-induced conditions. It is conceivable that CNV mutations affecting exon 8 may influence splicing efficiency, RNA processing, or other regulatory mechanisms—particularly in response to viral infection or inflammatory stress, such as SARS-CoV-2. Recent studies have shown that *MAPT* splicing is sensitive to cellular stress and may shift toward non-canonical isoform expression in disease contexts. Thus, our findings may reflect a stress-induced reactivation of exon 8 usage or an indirect regulatory consequence of structural variation at this locus. Although further experimental validation is needed, this observation—while intriguing—remains speculative and should be viewed as a preliminary signal that warrants replication in future studies.

A weak association was observed with exon 7, which could be attributed to the small sample size or the limited sensitivity of the neurocognitive assessment tools used. While memory impairment showed low statistical significance in relation to MAPT CNV mutations in exons 8 and 9, these findings may potentially correlate with memory decline in a larger sample or with more sensitive memory assessments.

*GRN* is another key gene implicated in FTLD [57]. *GRN*-related frontotemporal dementia (*GRN*-FTD) is inherited in an autosomal dominant manner, with nearly 95% of affected individuals having an affected parent [61]. Heterozygous mutations are found in approximately 5–25% of familial FTLD cases and 5% of sporadic FTLD cases [55]. The *GRN* gene, located on chromosome 17, is primarily associated with behavioral variant frontotemporal dementia (FTD), primary progressive aphasia (PPA), atypical parkinsonism, and corticobasal syndrome when mutations occur [61]. Of the 172 identified *GRN* mutations, 79 are considered pathogenic [62].

In our study, we examined five exons of the *GRN* gene (1, 3, 6, 10, 12). CNV mutations in *GRN* exon 6 and exon 12 showed the strongest associations with cognitive impairment. Regarding executive dysfunction, particularly in abstract thinking, we observed significant associations with CNV mutations in *GRN* exon 1, exon 6, and exon 12. However, there is a gap in the data that clearly links CNV mutations to disease development, and it remains unclear how combinations of multiple CNV mutations within a single gene might influence the overall mutagenic process. In our cohort, most participants, selected using a stratified sampling technique, were carriers of genes associated with EOAD and FTD, including *APP*, *PSEN1*, *PSEN2*, *MAPT*, and *GRN*. At this stage, it remains unclear whether the observed results are due to natural biological processes or if specific CNV mutations are directly responsible for cognitive impairment. It is yet to be determined how many of the participants will develop EOAD or FTD with cognitive impairment in the future. Currently, there is no national registry for diseases leading to cognitive impairment, which limits our ability to provide clear data on this issue and to explore potential ethnic differences.

While the role of viruses in dementia development remains debated, a meta-analysis suggests that viral exposure could be a potential risk factor for cognitive decline [40]. Herpes viruses, for instance, may contribute to the development of late-onset AD [63,64], potentially through mechanisms such as β-amyloidosis [65]. Herpes infection is particularly linked to executive function impairment, affecting regions like the orbitofrontal and anterior cingulate cortices [66], a finding that aligns with our study.

In a similar vein, over 50% of individuals with viral Hepatitis experience cognitive impairment [67], even in the absence of cirrhosis [68] or hepatic encephalopathy [43]. Several studies have highlighted that liver pathologies can impair the liver’s ability to eliminate beta-amyloid from the bloodstream [69], promoting its accumulation in the brain and potentially contributing to Alzheimer’s disease [60]. Individuals with chronic Hepatitis B and C are at an increased risk for deficits in executive function, mental processing speed, learning capacity, memory, complex attention, perceptual–motor skills, and social cognition [70]. Our research further suggests a significant link between Hepatitis B and linguistic impairment, particularly in object-naming ability. Additionally, seropositive Hepatitis A has been associated with diminished psychomotor speed [41], and our findings indicate a similar association with executive dysfunction, particularly in visuospatial abilities.

To address the question of causality between CNV mutations and neuronal vulnerability to stressors such as SARS-CoV-2 infection or other viral exposures, future hypothesis-generating research is warranted. One possible experimental direction involves in vitro models using neuronal cultures with gene-specific knockouts to assess differential responses to viral stress. While promising, this concept remains exploratory and would require validation through a series of preclinical steps. As a more immediate translational pathway, future efforts should focus on replicating our CNV findings in larger, ethnically diverse cohorts, characterizing the functional consequences of CNVs through transcriptomic or epigenetic profiling, and developing early detection tools (e.g., biomarker panels) for individuals at risk of cognitive decline. Additionally, cost-effectiveness studies of population-level genomic screening could help assess the feasibility of incorporating such approaches into precision medicine strategies for post-COVID-19 cognitive vulnerability.

### 3.2. Strengths and Limitations

#### 3.2.1. Strengths

A key strength of this study is the inclusion of the PHQ-9 to quantify depressive symptoms, which are known to influence subjective and objective measures of cognitive function. To address the potential confounding role of depressive symptoms in cognitive impairment, we included PHQ-9 scores as covariates in the adjusted regression models. The inclusion of PHQ-9 modestly attenuated some associations between CNVs and cognitive scores, particularly in RBANS subdomains, but key associations—especially involving *PSEN1* exon 9 and *MAPT* exon 2—remained statistically significant. This suggests that while depressive mood contributes to cognitive variability, the genetic associations observed may be at least partially independent of depression.

#### 3.2.2. Limitations

Our study cohort consists exclusively of individuals of Armenian nationality recruited from a single hospital complex, which limits the generalizability of our findings to broader populations. Genetic backgrounds, environmental factors, and healthcare access can vary significantly across different ethnic groups and geographical regions, potentially influencing the prevalence and impact of CNV mutations in the genes studied. Therefore, while our results provide valuable insights into the Armenian subpopulation, caution is warranted when extrapolating these findings to other ethnicities or populations. Future studies involving diverse cohorts from multiple geographic locations are essential to validate and expand upon our observations, ensuring broader applicability.

In addition, although two cognitive assessment tools—MoCA and RBANS—were utilized, our analysis prioritized MoCA due to its higher sensitivity to mild cognitive impairment and broader cross-cultural adaptability. The Armenian version of MoCA has been routinely applied in clinical practice, whereas RBANS, while domain-specific and comprehensive, poses challenges in non-English-speaking populations due to linguistic and normative limitations. Specifically, in the absence of Armenian national RBANS norms, we relied on cohort-specific median scores rather than standardized cutoffs, which may influence the interpretation of RBANS-derived results. This further underscores the need for culturally adapted and validated neuropsychological tools in future cognitive research in this population.

Moreover, the modest sample size (N = 162), relative to the high dimensionality of genetic and neurocognitive data, represents an additional limitation. Although our study identified statistically significant associations between specific CNV mutations and cognitive outcomes, the limited cohort size may reduce statistical power and increase the risk of both false positives and false negatives. For example, findings involving *MAPT* exon 8 and *PSEN1* exon 12 should be interpreted with restraint, given the wide confidence intervals and the exploratory nature of these analyses. Therefore, these findings should be interpreted with caution and considered exploratory. Future studies involving larger, independent cohorts with harmonized genetic and neurocognitive assessments are essential to validate these associations and further elucidate the role of CNVs in post-COVID-19 cognitive vulnerability.

## 4. Materials and Methods

### 4.1. Study Design

**Figure 4:**
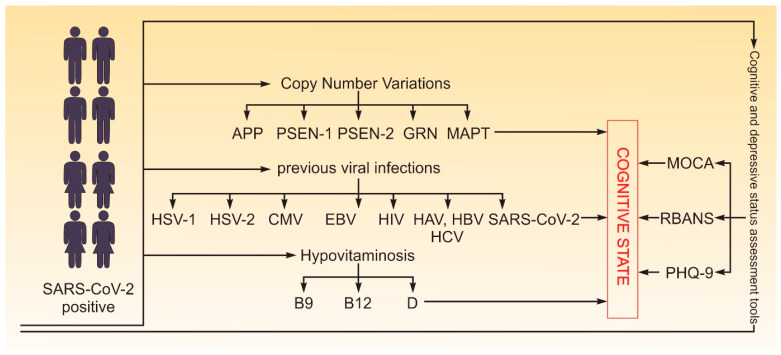
Study flow. In total, 162 participants (both male and female) aged 19 to 65 years with confirmed SARS-CoV-2 were tested for cognitive state with MoCA, RBANS and PHQ-9 (for depression), CNV mutations in genes associated with early-onset neurodegenerative diseases (*APP*, *PSEN1*, *PSEN2*, *GRN*, *MAPT*), hypovitaminosis (B12, B9, D), and viral infections (SARS-CoV-2, HSV-1, HSV-2, CMV, EBV, HIV, Hepatitis A, B, and C).

A case–control design was employed for this study. The case group consisted of individuals with an objective decline in cognitive function, while the control group included individuals with a normal cognitive state. Classification into case or control groups was based solely on current objective cognitive test results, not on self-reported history. Cognitive status was assessed based on performance in key mental domains, including perception, learning, memory, understanding, awareness, reasoning, judgment, attention, and language [71]. Although 28 participants (17%) reported subjective cognitive difficulties prior to COVID-19, none had undergone formal neuropsychological evaluation before infection. Due to the lack of pre-COVID-19 cognitive data, we did not treat this subgroup separately, and allocation was based on their current post-COVID-19 cognitive status. We analyzed cognitive function in relation to various risk factors for decline, including age, gender, CNV mutations in genes associated with early-onset neurodegenerative diseases (*APP*, *PSEN1*, *PSEN2*, *GRN*, *MAPT*), hypovitaminosis (B12, B9, D), previous viral infections (SARS-CoV-2, HSV-1, HSV-2, CMV, EBV, HIV, Hepatitis A, B, and C), and depression status. The study flow is depicted in Figure 4.

### 4.2. Participants

We recruited participants from the Heratsi Hospital Complex in Yerevan, Armenia, between January 2020 and August 2022. Data collection occurred during outpatient visits and included a single physical examination, laboratory tests, and cognitive and depression assessments. A total of 162 participants (both male and female) aged 19 to 65 years confirmed SARS-CoV-2 infection via RT-PCR or rapid antigen test were included. These participants had or did not have a prior history of cognitive impairment. Patients were excluded if they exhibited an impaired level of consciousness, required mechanical ventilation, or had severe organ failure during the COVID-19 pandemic. All participants provided informed consent to participate in this study and were of Armenian nationality.

### 4.3. Cognitive Assessments

Cognitive function was measured using the Repeatable Battery for the Assessment of Neuropsychological Status (RBANS) [33] and the Montreal Cognitive Assessment (MoCA) [52], with both total scores and domain-specific scores analyzed as primary outcome variables. The RBANS is a standardized neuropsychological battery designed to evaluate immediate memory, visuospatial/constructional abilities, language, attention, and delayed memory, whereas the MoCA is a brief screening tool used to detect mild cognitive impairment, assessing domains such as attention, executive function, memory, language, visuospatial skills, abstraction, calculation, and orientation. Both the RBANS and MoCA have been previously translated and culturally adapted for use in the Armenian language [72].

The results of both the MoCA and RBANS tests were converted into a nominal scale of positive or negative cognitive impairment status, based on the overall score. This transformation was made in line with the study’s aim—to determine the presence or absence of cognitive impairment rather than the level of impairment. A cutoff score of 26/30 was used for the MoCA test, with scores ≥ 26 indicating normal cognitive function and scores < 26 indicating impaired cognitive function [73]. For the RBANS test, a threshold of 90 points was selected, which varies ± 10 standard deviations (SDs) from the American norms (with a ±10–15 SD range allowed according to test guidelines) [18]. This threshold was chosen as more applicable for the Armenian population, based on our sample’s average scores and previous research findings [72].

### 4.4. Depression Assessment

Depression status was assessed using the PHQ-9 test, with scores of 0–4 indicating normal status and scores ≥ 5 indicating the presence of depression [74].

Clinical Interview: Participants also took part in a qualitative interview, which included a set of standardized questions about their experience with common post-COVID-19 symptoms, including fatigue, mood changes, and cognitive difficulties such as memory lapses, attention problems, and slowed thinking. Participants were asked to reflect on whether these symptoms were present before or emerged after their COVID-19 illness.

### 4.5. Genetic Analysis

The explanatory variables were measured using the SALSA Multiplex Ligation-Dependent Probe Amplification (MLPA) technique for the detection of CNVs. CNVs were identified as either homozygous or heterozygous deletions or duplications in the following genes: SALSA MLPA probemix P471-A1 for *PSEN1* (Exons 1–12), *PSEN2* (Exons 1–13), and *APP* (Exons 1–18) [75], and SALSA MLPA probemix P275-C3 for *MAPT* (Exons 1–14) and *GRN* (Exons 1, 3, 6, 10, 12) [76]. In the dataset, a value of 0 denotes a homozygous mutation, 1 indicates a heterozygous mutation, and 2 corresponds to individuals without CNVs in the respective exon.

### 4.6. Laboratory Measurements

The Electrochemiluminescence Immunoassay (ECLIA) method was used to analyze viral markers (HSV-1, HSV-2, CMV, EBV, HIV, SARS-CoV-2, Hepatitis A, B, and C) and detect deficiencies in vitamins D, B12, and B9 from blood samples. Hypovitaminosis was defined by the following thresholds: vitamin D—<30 ng/mL; vitamin B12—<191 pg/mL; and vitamin B9—<3.1 ng/mL. The following thresholds were applied for viral infections—SARS-CoV-2 IgG: >1.0 COI; HSV-1: <0.9 COI; HSV-2: <0.9 COI; CMV: <0.5 U/mL; EBV: <1.0 COI; HIV: <0.9 COI; Hepatitis A: >1.0 COI; Hepatitis B: <0.9 COI; Hepatitis C: <0.9 COI. Tests were properly calibrated and regularly controlled according to the manufacturer’s recommendations (Roche Diagnostics GmbH, Mannheim, Germany).

### 4.7. Statistical Methods

All statistical analyses were performed using R version 4.3.2 (R Core Team, 2023) within the RStudio environment (version 2023.09.1+494). A significance level of *p* < 0.05 was applied to all tests unless otherwise specified. The following approaches were employed:

a. Non-Parametric Methods

For continuous variables, Spearman’s rank correlation coefficient was used to assess correlations. Group comparisons across independent groups were evaluated using the Mann-Whitney U test or Kruskal–Wallis test depending on the number of groups. Male sex and a low level of AHAV were used as a reference level when performing a Mann–Whitney test.

b. Categorical Variables

Associations between categorical variables were analyzed using the chi-square test of independence.

c. Gene–Score Associations

The association between a gene and cognitive function scores, including the MoCA score, RBANS score, and self-reported cognitive impairment scores, was analyzed using model–submodel tests. In these models, cognitive scores were treated as dependent variables, while individual exons (presence/absence of mutation) were modeled as independent categorical predictors. Using the model–submodel approach in each test, we compared a full model containing all relevant exons and a trivial model, containing only an intercept.

d. Multiple Comparison Adjustment

For multiple hypothesis testing within each feature group (anthropometric measurements, depression scores, genetic variables, vitamins, and viruses), Benjamini–Hochberg (BH) correction was applied to control the false discovery rate.

e. Multiple Regression for Gene Validation

Significant genes identified in the initial analyses were further validated using multiple regression models. In these models, all relevant exons were included as independent categorical variables, while cognitive function scores were used as the dependent variable. A model–submodel comparison test was performed for each exon to obtain a single *p*-value per exon, thereby maximizing statistical power. Additionally, we assessed contrasts highlighting differences between categories 1 and 2 compared to category 0, which was treated as the reference. From this model, we derived effect sizes corresponding to different heterozygous and homozygous mutation statuses.

## 5. Conclusions

The significant prevalence of CNV mutations in the *APP*, *PSEN1*, *PSEN2*, *MAPT*, and GRN genes within the Armenian population may have potential pathogenic impacts on cognitive functions. We identified associations with cognitive impairment specifically in the CNV mutations of the PSEN1 (exons 1, 9, 12), GRN (exons 1, 6, 12), and MAPT (exons 2, 8) genes. Although genetic mutations do not directly influence self-reported cognitive deterioration, post-COVID-19 cognitive complaints are primarily associated with depression. Cognitive impairment in our cohort is also correlated with factors such as age, gender, depressive mood, viral infections (HSV1, HSV2, HAV, and HBV), and folic acid insufficiency. The findings of this study are primarily applicable to the subpopulation of patients exposed to SARS-CoV-2. However, further research is needed to assess the broader applicability of these results to other populations or contexts.

## Figures and Tables

**Table 1:** Statistical associations between *PSEN1* exons and cognitive impairment domains identified from MoCA scores. (**A**) associations between *PSEN1* and MoCA delayed recall; (**B**) associations between *PSEN1* and MoCA total score.

**A. Associations between *PSEN1* and MoCA delayed recall**							
**a—model–submodel test**							
**Exon**	**Df**	**Sum of Sq**	**RSS**	**AIC**	**F Value**	***p*** **Value**	
*‘PSEN1* *Exon 1’*	2	32.00	450	190	5.100	0.0070	
‘*PSEN1* *Exon 2’*	1	9.50	430	180	3.100	0.0820	
‘*PSEN1* *Exon 3’*	1	4.00	430	180	1.300	0.2600	
‘*PSEN1* *Exon 5’*	1	4.80	430	180	1.500	0.2200	
‘*PSEN1* *Exon 6’*	2	0.18	420	180	0.029	0.9700	
‘*PSEN1* *Exon 7’*	1	4.00	430	180	1.300	0.2600	
‘*PSEN1* *Exon 8’*	1	4.80	430	180	1.500	0.2200	
‘*PSEN1* *Exon 9’*	1	11.00	430	180	3.500	0.0650	
‘*PSEN1* *Exon 10’*	1	5.70	430	180	1.900	0.1800	
‘*PSEN1* *Exon 12’*	2	31.00	450	190	5.000	0.0079	
**b—linear regression with dummy variables**							
**Ex.**	**Term**	**est.**	**SE**	**Statistic**	***p*** **Value**	**conf.low**	**conf.high**
1	(Intercept)	2.80	4.60	6.00 × 10^−1^	0.547	−6.30	12.00
2	‘*PSEN1* *Exon 1’1*	−1.50	0.49	−3.00	0.004	−2.40	−0.48
3	‘*PSEN1* *Exon 1’2*	−1.60	0.51	−3.10	0.002	−2.60	−0.57
4	‘*PSEN1* *Exon 2’2*	−1.10	0.61	−1.80	0.082	−2.30	0.14
5	‘*PSEN1* *Exon 3’2*	2.40	2.10	1.10	0.258	−1.80	6.50
6	‘*PSEN1* *Exon 5’2*	−2.30	1.90	−1.20	0.217	−6.00	1.40
7	‘*PSEN1* *Exon 6’1*	1.60 × 10^−1^	2.30	7.20 × 10^−2^	0.943	−4.30	4.60
8	‘*PSEN1* *Exon 6’2*	4.90 × 10^−15^	2.20	2.30 × 10^−15^	1.000	−4.30	4.30
9	‘*PSEN1* *Exon 7’2*	−9.40 × 10^−1^	0.83	−1.10	0.257	−2.60	0.70
10	‘*PSEN1* *Exon 8’2*	−2.30	1.90	−1.20	0.217	−6.00	1.40
11	‘*PSEN1* *Exon 9’2*	3.50	1.90	1.90	0.065	−0.22	7.10
12	‘*PSEN1* *Exon 10’2*	2.60	1.90	1.40	0.176	−1.20	6.30
13	‘*PSEN1* *Exon 12’1*	−2.40	1.40	−1.80	0.075	−5.10	0.25
14	‘*PSEN1* *Exon 12’2*	−7.20 × 10^−1^	1.30	−5.70 × 10^−1^	0.570	−3.20	1.80
**B. Associations between *PSEN1* and MoCA total score**							
**a—model–submodel test**							
**Exon**	**Df**	**Sum of Sq**	**RSS**	**AIC**	**F Value**	***p*** **Value**	
‘*PSEN1* *Exon 1’*	2	66.00	1500	370	3.000	0.051	
‘*PSEN1* *Exon 2’*	1	26.00	1500	370	2.400	0.120	
‘*PSEN1* *Exon 3’*	1	0.83	1500	370	0.077	0.780	
‘*PSEN1* *Exon 5’*	1	11.00	1500	370	1.000	0.320	
‘*PSEN1* *Exon 6’*	2	2.00	1500	370	0.094	0.910	
‘*PSEN1* *Exon 7’*	1	3.00	1500	370	0.270	0.600	
‘*PSEN1* *Exon 8’*	1	11.00	1500	370	1.000	0.320	
‘*PSEN1* *Exon 9’*	1	55.00	1500	370	5.100	0.026	
‘*PSEN1* *Exon 10’*	1	17.00	1500	370	1.500	0.220	
‘*PSEN1* *Exon 12’*	2	80.00	1600	370	3.700	0.027	
**b—linear regression with dummy variables**							
**Ex.**	**Term**	**est.**	**std.error**	**Statistic**	***p*** **Value**	**conf.low**	**conf.high**
1	(Intercept)	33.00	8.60	3.80	<0.001	16.00	50.00
2	‘*PSEN1* *Exon 1’1*	−0.23	0.92	−0.25	0.800	−2.10	1.60
3	‘*PSEN1* *Exon 1’2*	−1.70	0.95	−1.70	0.085	−3.50	0.23
4	‘*PSEN1* *Exon 2’2*	−1.80	1.10	−1.60	0.122	−4.00	0.48
5	‘*PSEN1* *Exon 3’2*	1.10	3.90	0.28	0.782	−6.60	8.80
6	‘*PSEN1* *Exon 5’2*	−3.50	3.50	−1.00	0.320	−10.00	3.40
7	‘*PSEN1* *Exon 6’1*	0.55	4.20	0.13	0.897	−7.80	8.90
8	‘*PSEN1* *Exon 6’2*	1.00	4.00	0.25	0.804	−7.00	9.00
9	‘*PSEN1* *Exon 7’2*	−0.81	1.50	−0.52	0.602	−3.90	2.20
10	‘*PSEN1* *Exon 8’2*	−3.50	3.50	−1.00	0.320	−10.00	3.40
11	‘*PSEN1* *Exon 9’2*	7.90	3.50	2.30	0.026	0.98	15.00
12	‘*PSEN1* *Exon 10’2*	−4.40	3.50	−1.20	0.217	−11.00	2.60
13	‘*PSEN1* *Exon 12’1*	−4.90	2.50	−1.90	0.056	−9.90	0.12
14	‘*PSEN1* *Exon 12’2*	−2.30	2.40	−0.98	0.329	−7.00	2.40

**Table 2:** Statistical associations between *MAPT* exons and cognitive impairment domains identified from MoCA scores. (**A**) associations between *MAPT* and MoCA delayed recall; (**B**) associations between PSEN1 and MoCA total score.

**A. Associations between *MAPT* and MoCA delayed recall**							
**a—model–submodel test**							
**Exon**	**Df**	**Sum of Sq**	**RSS**	**AIC**	**F Value**	***p*** **Value**	
‘*MAPT* *Exon 1’*	2	6.100	420	190	0.9400	0.390	
‘*MAPT* *Exon 2’*	2	28.000	440	200	4.4000	0.014	
‘*MAPT* *Exon 3’*	0	0.000	410	190			
‘*MAPT* *Exon 4’*	0	0.000	410	190			
‘*MAPT* *Exon 5’*	1	2.900	420	190	0.9200	0.340	
‘*MAPT Exon 6’*	2	0.049	410	190	0.0076	0.990	
‘*MAPT* *Exon 7’*	1	0.910	410	190	0.2900	0.590	
‘*MAPT Exon 8’*	2	18.000	430	200	2.9000	0.060	
‘*MAPT* *Exon 9’*	1	9.800	420	200	3.1000	0.083	
‘*MAPT* *Exon 10’*	1	8.200	420	200	2.6000	0.110	
‘*MAPT* *Exon 11’*	1	3.600	420	190	1.1000	0.290	
‘*MAPT* *Exon 12’*	2	0.750	410	190	0.1200	0.890	
‘*MAPT* *Exon 13’*	2	9.400	420	190	1.5000	0.230	
‘*MAPT* *Exon 14’*	2	2.700	420	190	0.4300	0.650	
**b—linear regression with dummy variables**							
**Ex.**	**Term**	**Est.**	**std.Error**	**Statistic**	***p*** **Value**	**conf.low**	**conf.high**
1	(Intercept)	−2.500	4.60	−0.560	0.579	−12.00	6.50
2	‘*MAPT* *Exon 1’1*	0.380	0.36	1.000	0.300	−0.34	1.10
3	‘*MAPT* *Exon 1’2*	0.540	0.51	1.100	0.291	−0.47	1.50
4	‘*MAPT* *Exon 2’1*	1.400	1.20	1.200	0.244	−0.99	3.80
5	‘*MAPT* *Exon 2’2*	2.700	1.10	2.400	0.017	0.49	4.80
6	‘*MAPT* *Exon 3’2*	1.600	2.80	0.570	0.568	−4.00	7.20
8	‘*MAPT* *Exon 5’2*	1.800	1.90	0.960	0.340	−1.90	5.50
9	‘*MAPT Exon 6’1*	0.094	1.10	0.088	0.930	−2.00	2.20
10	‘*MAPT Exon 6’2*	0.034	1.30	0.026	0.979	−2.50	2.50
11	‘*MAPT* *Exon 7’2*	0.590	1.10	0.530	0.594	−1.60	2.80
12	‘*MAPT Exon 8’1*	−3.500	1.70	−2.000	0.043	−7.00	−0.12
13	‘*MAPT Exon 8’2*	−2.800	1.70	−1.600	0.108	−6.10	0.61
14	‘*MAPT* *Exon 9’2*	1.100	0.60	1.700	0.083	−0.14	2.30
15	‘*MAPT* *Exon 10’2*	−0.610	0.38	−1.600	0.112	−1.40	0.14
16	‘*MAPT* *Exon 11’2*	−0.530	0.50	−1.100	0.289	−1.50	0.45
17	‘*MAPT* *Exon 12’1*	−0.650	2.00	−0.330	0.744	−4.60	3.30
18	‘*MAPT* *Exon 12’2*	−0.920	2.10	−0.440	0.661	−5.00	3.20
19	‘*MAPT* *Exon 13’1*	3.200	1.90	1.700	0.095	−0.57	7.00
20	‘*MAPT* *Exon 13’2*	2.900	1.90	1.500	0.129	−0.86	6.70
21	‘*MAPT* *Exon 14’1*	−1.100	2.20	−0.500	0.615	−5.30	3.20
22	‘*MAPT* *Exon 14’2*	−1.400	2.20	−0.640	0.522	−5.60	2.90
**B. Associations between *MAPT* and MoCA total score**							
**a—model–submodel test**							
**Exon**	**Df**	**Sum of Sq**	**RSS**	**AIC**	**F Value**	***p*** **Value**	
‘*MAPT* *Exon 1’*	2	7.9	1400	370	0.37	0.690	
‘*MAPT* *Exon 2’*	2	37.0	1400	370	1.70	0.180	
‘*MAPT* *Exon 5’*	1	25.0	1400	380	2.40	0.130	
‘*MAPT Exon 6’*	2	10.0	1400	370	0.49	0.610	
‘*MAPT* *Exon 7’*	1	33.0	1400	380	3.00	0.083	
‘*MAPT Exon 8’*	2	75.0	1500	380	3.50	0.033	
‘*MAPT* *Exon 9’*	1	14.0	1400	370	1.30	0.250	
‘*MAPT* *Exon 10’*	1	6.8	1400	370	0.63	0.430	
‘*MAPT* *Exon 11’*	1	8.3	1400	370	0.78	0.380	
‘*MAPT* *Exon 12’*	2	17.0	1400	370	0.77	0.460	
‘*MAPT* *Exon 13’*	2	40.0	1400	380	1.90	0.160	
‘*MAPT* *Exon 14’*	2	39.0	1400	380	1.80	0.160	
**b—linear regression with dummy variables**							
**Ex.**	**term**	**Est.**	**std.error**	**statistic**	***p*** **.value**	**conf.low**	**conf.high**
1	(Intercept)	28.00	8.40	3.30	0.001	11.00	44.00
2	‘*MAPT* *Exon 1’1*	−0.18	0.66	−0.26	0.792	−1.50	1.10
3	‘*MAPT* *Exon 1’2*	0.70	0.93	0.76	0.448	−1.10	2.50
4	*‘MAPT* *Exon 2’1*	1.70	2.20	0.78	0.436	−2.70	6.10
5	*‘MAPT* *Exon 2’2*	3.10	2.00	1.50	0.125	−0.87	7.10
6	*‘MAPT* *Exon 3’2*	−11.00	5.20	−2.00	0.044	−21.00	−0.27
7	*‘MAPT* *Exon 4’2*						
8	*‘MAPT* *Exon 5’2*	5.30	3.40	1.50	0.125	−1.50	12.00
9	*‘* *MAPT Exon 6* *’* *1*	1.70	2.00	0.90	0.372	−2.10	5.60
10	*‘* *MAPT Exon 6* *’* *2*	2.30	2.30	0.98	0.328	−2.30	6.90
11	*‘MAPT* *Exon 7’2*	3.50	2.00	1.70	0.083	−0.47	7.60
12	*‘* *MAPT Exon 8* *’* *1*	−7.20	3.20	−2.30	0.023	−13.00	−1.00
13	*‘* *MAPT Exon 8* *’* *2*	−5.70	3.10	−1.80	0.068	−12.00	0.44
14	*‘MAPT* *Exon 9’2*	1.30	1.10	1.20	0.248	−0.9.00	3.50
15	*‘MAPT* *Exon 10’2*	−0.55	0.69	−0.80	0.428	−1.9.00	0.82
16	*‘MAPT* *Exon 11’2*	−0.80	0.91	−0.88	0.379	−2.60	0.99
17	*‘MAPT* *Exon 12’1*	−1.70	3.60	−0.48	0.633	−9.00	5.50
18	*‘MAPT* *Exon 12’2*	−3.40	3.80	−0.88	0.379	−11.00	4.20
19	*‘MAPT* *Exon 13’1*	6.80	3.50	1.90	0.055	−0.16	14.00
20	*‘MAPT* *Exon 13’2*	6.50	3.50	1.90	0.064	−0.38	13.00
21	*‘MAPT* *Exon 14’1*	−3.40	3.90	−0.87	0.384	−11.00	4.30
22	*‘MAPT* *Exon 14’2*	−4.60	3.90	−1.20	0.240	−12.00	3.10

**Table 3:** Statistical associations between *GRN* exons and cognitive impairment domains identified from MoCA scores. (**A**) associations between *MAPT* and MoCA delayed recall; (**B**) associations between *PSEN1* and MoCA total score.

**A. Associations between *GRN* and MoCA delayed recall**							
**a—model–submodel test**							
**Exon**	**Df**	**Sum of Sq**	**RSS**	**AIC**	**F Value**	***p* Value**	
*‘GRN* *Exon 1’*	1	0.6200	18	−300	5.000	0.0270	
*‘GRN* *Exon 3’*	1	0.0032	17	−310	0.026	0.8700	
*‘GRN* *Exon 6’*	2	1.5000	19	−300	6.100	0.0029	
*‘GRN* *Exon 10’*	2	0.2200	18	−310	0.880	0.4200	
*‘GRN* *Exon 12’*	2	0.7400	18	−300	3.000	0.0530	
**b—linear regression with dummy variables**							
**Ex.**	**Term**	**est.**	**std.Error**	**Statistic**	***p* Value**	**conf.low**	**conf.high**
1	(Intercept)	1.300	0.22	5.90	<0.001	0.860	1.700
2	*‘GRN* *Exon 1’2*	0.390	0.17	2.20	0.027	0.045	0.730
3	*‘GRN* *Exon 3’2*	−0.017	0.11	−0.16	0.872	−0.230	0.190
4	*‘GRN* *Exon 6’1*	0.480	0.16	3.00	0.004	0.160	0.810
5	*‘GRN* *Exon 6’2*	0.280	0.18	1.60	0.120	−0.075	0.640
6	*‘GRN* *Exon 10’1*	−0.063	0.11	−0.56	0.575	−0.280	0.160
7	*‘GRN* *Exon 10’2*	0.087	0.16	0.53	0.598	−0.240	0.410
8	*‘GRN* *Exon 12’1*	−0.190	0.14	−1.30	0.194	−0.470	0.096
9	*‘GRN* *Exon 12’2*	0.073	0.18	0.40	0.691	−0.290	0.430
**B. Associations between *GRN* and MoCA total score**							
**a—model–submodel test**							
**Exon**	**Df**	**Sum of Sq**	**RSS**	**AIC**	**F Value**	***p* Value**	
*‘GRN* *Exon 1’*	1	0.35	1500	360	0.033	0.86000	
*‘GRN* *Exon 3’*	1	16.00	1500	360	1.600	0.21000	
*‘GRN* *Exon 6’*	2	200.00	1700	380	9.400	0.00015	
*‘GRN* *Exon 10’*	2	56.00	1500	360	2.600	0.07500	
*‘GRN* *Exon 12’*	2	150.00	1600	370	7.000	0.00130	
**b—linear regression with dummy variables**							
**Ex.**	**Term**	**Est.**	**std.error**	**Statistic**	***p* Value**	**conf.low**	**conf.high**
1	(Intercept)	22.00	2.00	11.00	<0.001	18.00	26.00
2	*‘GRN* *Exon 1’2*	0.29	1.60	0.18	0.856	−2.90	3.40
3	*‘GRN* *Exon 3’2*	1.20	0.98	1.20	0.214	−0.71	3.20
4	*‘GRN* *Exon 6’1*	3.90	1.50	2.60	0.010	0.95	6.90
5	*‘GRN* *Exon 6’2*	0.73	1.70	0.44	0.662	−2.60	4.10
6	*‘GRN* *Exon 10’1*	2.20	1.00	2.10	0.037	0.14	4.20
7	*‘GRN* *Exon 10’2*	3.20	1.50	2.10	0.039	0.16	6.20
8	*‘GRN* *Exon 12’1*	−3.60	1.30	−2.70	0.007	−6.20	−0.97
9	*‘GRN* *Exon 12’2*	−0.55	1.70	−0.32	0.746	−3.90	2.80

## Data Availability

Data can be made available by the corresponding author upon reasonable request.

## Supplementary Materials

The following supporting information can be downloaded at: https://www.mdpi.com/article/10.3390/ijms26146965/s1.
