# Supplementary material for: Genetic Correlates of Presenile Dementia and Cognitive Decline in the Armenian Population Following COVID-19: A Case-Control Study
Source: Int J Mol Sci. 2025 Jul 20;26(14):6965. doi: 10.3390/ijms26146965 (PMC12295909; doi:10.3390/ijms26146965)
Supplement: Supplementary file 1 [file ijms-26-06965-s001.zip › Supplementary_ Table S1_ Descriptive statistics.pdf]

Table S1: Descriptive statistics

Variable	N	N = 162[1]
**Age**	162	42 (13)
**Sex**	162	
F		106 (65%)
M		56 (35%)
**CMV IgG**	125	
high		121 (97%)
low		4 (3.2%)
Unknown		37
**HIVCOMPT**	149	
high		1 (0.7%)
low		148 (99%)
Unknown		13
**HSV-1**	131	
high		88 (67%)
low		43 (33%)
Unknown		31
**HSV-2**	129	
high		9 (7.0%)
low		120 (93%)
Unknown		33
**A-HCVII**	142	
high		1 (0.7%)
low		141 (99%)
Unknown		20
**EBVEBNA IgG**	134	
high		120 (90%)
low		14 (10%)
Unknown		28
**HBSAGII**	148	
high		5 (3.4%)
low		143 (97%)

Unknown		14
**AHAV 2**	129	
high		22 (17%)
low		107 (83%)
Unknown		33
**ACOV2 IgG**	148	
high		142 (96%)
low		6 (4.1%)
Unknown		14
**B12 II**	162	
high		9 (5.6%)
low		22 (14%)
normal		131 (81%)
**VitD II**	162	
high		1 (0.6%)
low		78 (48%)
normal		83 (51%)
**FOL III**	162	
high		3 (1.9%)
low		40 (25%)
normal		119 (73%)
**PHQ-9**	161	6.9 (5.4)
Unknown		1
**MOCA visuospecial/executive**	161	4.44 (0.81)
Unknown		1
**MOCA naming**	161	2.85 (0.41)
Unknown		1
**MOCA attention**	161	5.31 (1.07)
Unknown		1
**MOCA language**	161	2.25 (0.77)
Unknown		1
**MOCA abstraction**	161	1.88 (0.36)
Unknown		1
**MOCA Delayed recall**	161	2.57 (1.87)
Unknown		1
**MOCA orientation**	161	5.88 (0.38)

Unknown		1
**MOCA TOTAL**	161	25.6 (3.4)
Unknown		1
**RBANS Immediate Memory**	162	95 (17)
**RBANS Visuospatial Constructional**	162	103 (16)
**RBANS Language**	162	88 (17)
**RBANS Attention**	162	85 (16)
**RBANS Delayed Memory**	162	94 (16)
**RBANS Total Scale of Index Scores**	162	90 (13)
**Memory impairment after COVID-19**	162	92 (57%)
**MAPT Exon 1**	151	
0		93 (62%)
1		40 (26%)
2		18 (12%)
Unknown		11
**MAPT Exon 2**	151	
0		4 (2.6%)
1		10 (6.6%)
2		137 (91%)
Unknown		11
**MAPT Exon 3**	151	
1		1 (0.7%)
2		150 (99%)
Unknown		11
**MAPT Exon 4**	151	
1		1 (0.7%)
2		150 (99%)
Unknown		11
**MAPT Exon 5**	151	
1		2 (1.3%)
2		149 (99%)
Unknown		11
**MAPT Exon 6**	151	
0		7 (4.6%)
1		134 (89%)
2		10 (6.6%)

Unknown		11
**MAPT Exon 7**	151	
1		4 (2.6%)
2		147 (97%)
Unknown		11
**MAPT Exon 8**	151	
0		2 (1.3%)
1		23 (15%)
2		126 (83%)
Unknown		11
**MAPT Exon 9**	151	
1		17 (11%)
2		134 (89%)
Unknown		11
**MAPT Exon 10**	151	
1		64 (42%)
2		87 (58%)
Unknown		11
**MAPT Exon 11**	151	
1		37 (25%)
2		114 (75%)
Unknown		11
**MAPT Exon 12**	151	
0		1 (0.7%)
1		143 (95%)
2		7 (4.6%)
Unknown		11
**MAPT Exon 13**	151	
0		1 (0.7%)
1		18 (12%)
2		132 (87%)
Unknown		11
**MAPT Exon 14**	151	
0		1 (0.7%)
1		30 (20%)

2		120 (79%)
Unknown		11
**GRN Exon 1**	151	
1		5 (3.3%)
2		146 (97%)
Unknown		11
**GRN Exon 3**	151	
1		17 (11%)
2		134 (89%)
Unknown		11
**GRN Exon 6**	151	
0		7 (4.6%)
1		108 (72%)
2		36 (24%)
Unknown		11
**GRN Exon 10**	151	
0		12 (7.9%)
1		128 (85%)
2		11 (7.3%)
Unknown		11
**GRN Exon 12**	151	
0		12 (7.9%)
1		120 (79%)
2		19 (13%)
Unknown		11
**APP Upstream**	151	
0		20 (13%)
1		58 (38%)
2		73 (48%)
Unknown		11
**APP Exon 1**	151	
0		96 (64%)
1		49 (32%)
2		6 (4.0%)
Unknown		11

**APP Intron 1**	151	
1		2 (1.3%)
2		149 (99%)
Unknown		11
**APP Exon 2**	151	
2		151 (100%)
Unknown		11
**APP Exon 3**	151	
1		4 (2.6%)
2		147 (97%)
Unknown		11
**APP Exon 4**	151	
1		31 (21%)
2		120 (79%)
Unknown		11
**APP Exon 5**	151	
1		2 (1.3%)
2		149 (99%)
Unknown		11
**APP Exon 6**	151	
1		3 (2.0%)
2		148 (98%)
Unknown		11
**APP Exon 7**	151	
1		3 (2.0%)
2		148 (98%)
Unknown		11
**APP Exon 8**	151	
1		7 (4.6%)
2		144 (95%)
Unknown		11
**APP Exon 9**	151	
2		151 (100%)
Unknown		11
**APP Exon 10**	151	

2		151 (100%)
Unknown		11
**APP Exon 11**	151	
2		151 (100%)
Unknown		11
**APP Exon 12**	151	
1		1 (0.7%)
2		150 (99%)
Unknown		11
**APP Exon 13**	151	
1		2 (1.3%)
2		149 (99%)
Unknown		11
**APP Exon 14**	151	
1		5 (3.3%)
2		146 (97%)
Unknown		11
**APP Exon 15**	151	
0		1 (0.7%)
1		18 (12%)
2		132 (87%)
Unknown		11
**APP Exon 16**	151	
1		13 (8.6%)
2		138 (91%)
Unknown		11
**APP Exon 17**	151	
1		65 (43%)
2		86 (57%)
Unknown		11
**APP Exon 18**	151	
1		1 (0.7%)
2		150 (99%)
Unknown		11
**PSEN1 Exon 1**	151	

0		17 (11%)
1		77 (51%)
2		57 (38%)
Unknown		11
**PSEN1 Exon 2**	151	
1		13 (8.6%)
2		138 (91%)
Unknown		11
**PSEN1 Exon 3**	151	
1		1 (0.7%)
2		150 (99%)
Unknown		11
**PSEN1 Exon 4**	151	
2		151 (100%)
Unknown		11
**PSEN1 Exon 5**	151	
1		1 (0.7%)
2		150 (99%)
Unknown		11
**PSEN1 Exon 6**	151	
0		2 (1.3%)
1		10 (6.6%)
2		139 (92%)
Unknown		11
**PSEN1 Exon 7**	151	
1		6 (4.0%)
2		145 (96%)
Unknown		11
**PSEN1 Exon 8**	151	
1		3 (2.0%)
2		148 (98%)
Unknown		11
**PSEN1 Exon 9**	151	
1		1 (0.7%)
2		150 (99%)

Unknown		11
**PSEN1 Exon 10**	151	
1		1 (0.7%)
2		150 (99%)
Unknown		11
**PSEN1 Exon 11**	151	
2		151 (100%)
Unknown		11
**PSEN1 Exon 12**	151	
0		2 (1.3%)
1		17 (11%)
2		132 (87%)
Unknown		11
**PSEN2 Exon 1**	151	
0		127 (84%)
1		24 (16%)
Unknown		11
**PSEN2 Exon 2**	151	
0		39 (26%)
1		95 (63%)
2		17 (11%)
Unknown		11
**PSEN2 Exon 3**	151	
1		17 (11%)
2		134 (89%)
Unknown		11
**PSEN2 Exon 4**	151	
1		8 (5.3%)
2		143 (95%)
Unknown		11
**PSEN2 Exon 5**	151	
1		19 (13%)
2		132 (87%)
Unknown		11
**PSEN2 Exon 6**	151	

1		75 (50%)
2		76 (50%)
Unknown		11
**PSEN2 Exon 7**	151	
1		24 (16%)
2		127 (84%)
Unknown		11
**PSEN2 Exon 8**	151	
1		23 (15%)
2		128 (85%)
Unknown		11
**PSEN2 Exon 9**	151	
0		1 (0.7%)
1		84 (56%)
2		66 (44%)
Unknown		11
**PSEN2 Exon 10**	151	
1		60 (40%)
2		91 (60%)
Unknown		11
**PSEN2 Exon 11**	151	
0		2 (1.3%)
1		103 (68%)
2		46 (30%)
Unknown		11
**PSEN2 Exon 12**	151	
1		101 (67%)
2		50 (33%)
Unknown		11
**PSEN2 Exon 13**	151	
1		90 (60%)
2		61 (40%)
Unknown		11

[1]Mean (SD); n (%)
