# Supplementary material for: Genetic Correlates of Presenile Dementia and Cognitive Decline in the Armenian Population Following COVID-19: A Case-Control Study
Source: Int J Mol Sci. 2025 Jul 20;26(14):6965. doi: 10.3390/ijms26146965 (PMC12295909; doi:10.3390/ijms26146965)
Supplement: Supplementary file 1 [file ijms-26-06965-s001.zip › Supplementary_Table S2_ Genes statistics.pdf]

Impairment	Group	Feature	Method	Test output	p-value	p-value adjusted
MOCA Delayed recall	Demography	Age	Spearman correlation	estimate = -0.3; statistic = 9e+05; p.value = 0.00012; method = Spearman's rank correlation rho; alternative = two.sided	0.00012	0.00024
		Sex	KW/MW Test	estimate = 8.5e-05; statistic = 3000; p.value = 0.8; conf.low = -6.1e-05; conf.high = 1; method = Wilcoxon rank sum test with continuity correction; alternative = two.sided	0.8	0.8
	Depression	PHQ-9	Spearman correlation	estimate = -0.072; statistic = 750000; p.value = 0.37; method = Spearman's rank correlation rho; alternative = two.sided	0.366	0.366
	Genes	APP	GLM	df.residual = 130; residual.deviance = 450	0.234	0.292
		GRN	GLM	df.residual = 140; residual.deviance = 480	0.122	0.204
		MAPT	GLM	df.residual = 130; residual.deviance = 410	0.0389	0.0972
		PSEN1	GLM	df.residual = 140; residual.deviance = 420	0.00399	0.0199
		PSEN2	GLM	df.residual = 130; residual.deviance = 490	0.875	0.875
	Viruses	ACOV2 IgG	KW/MW Test	estimate = 1; statistic = 540; p.value = 0.25; conf.low = -6.1e-05; conf.high = 3; method = Wilcoxon rank sum test with continuity correction; alternative = two.sided	0.246	0.43
		AHAV 2	KW/MW Test	estimate = 1; statistic = 1400; p.value = 0.073; conf.low = -5.4e-05; conf.high = 2; method = Wilcoxon rank sum test with continuity correction; alternative = two.sided	0.0732	0.43
		CMV IgG	KW/MW Test	estimate = -4.3e-05; statistic = 210; p.value = 0.71; conf.low = -2; conf.high = 2; method =	0.708	0.708

Impairment	Group	Feature	Method	Test output	p-value	p-value adjusted
				Wilcoxon rank sum test with continuity correction; alternative = two.sided		
		EBVEBNA IgG	KW/MW Test	estimate = 5.2e-05; statistic = 910; p.value = 0.58; conf.low = -1; conf.high = 1; method = Wilcoxon rank sum test with continuity correction; alternative = two.sided	0.581	0.708
		HBSAGII	KW/MW Test	estimate = 4.6e-05; statistic = 400; p.value = 0.67; conf.low = -1; conf.high = 3; method = Wilcoxon rank sum test with continuity correction; alternative = two.sided	0.668	0.708
		HSV-1	KW/MW Test	estimate = -1.7e-05; statistic = 1600; p.value = 0.24; conf.low = -1; conf.high = 6.2e-05; method = Wilcoxon rank sum test with continuity correction; alternative = two.sided	0.243	0.43
		HSV-2	KW/MW Test	estimate = -1; statistic = 400; p.value = 0.21; conf.low = -2; conf.high = 1; method = Wilcoxon rank sum test with continuity correction; alternative = two.sided	0.21	0.43
	Vitamins	B12 II	KW/MW Test	statistic = 0.1; p.value = 0.95; parameter = 2; method = Kruskal-Wallis rank sum test	0.949	0.949
		FOL III	KW/MW Test	statistic = 6; p.value = 0.05; parameter = 2; method = Kruskal-Wallis rank sum test	0.0499	0.15
		VitD II	KW/MW Test	statistic = 0.34; p.value = 0.84; parameter = 2; method = Kruskal-Wallis rank sum test	0.844	0.949
MOCA TOTAL	Demography	Age	Spearman correlation	estimate = -0.26; statistic = 880000; p.value = 0.00072; method = Spearman's rank correlation rho; alternative = two.sided	0.000716	0.00143

Impairment	Group	Feature	Method	Test output	p-value	p-value adjusted
		Sex	KW/MW Test	estimate = -2.6e-05; statistic = 2800; p.value = 0.56; conf.low = -1; conf.high = 1; method = Wilcoxon rank sum test with continuity correction; alternative = two.sided	0.563	0.563
	Depression	PHQ-9	Spearman correlation	estimate = -0.021; statistic = 710000; p.value = 0.8; method = Spearman's rank correlation rho; alternative = two.sided	0.796	0.796
	Genes	APP	GLM	df.residual = 130; residual.deviance = 1400	0.0909	0.114
		GRN	GLM	df.residual = 140; residual.deviance = 1500	0.00319	0.016
		MAPT	GLM	df.residual = 130; residual.deviance = 1400	0.0362	0.0603
		PSEN1	GLM	df.residual = 140; residual.deviance = 1500	0.0276	0.0603
		PSEN2	GLM	df.residual = 130; residual.deviance = 1600	0.516	0.516
	Viruses	ACOV2 IgG	KW/MW Test	estimate = 2; statistic = 570; p.value = 0.16; conf.low = -1; conf.high = 4; method = Wilcoxon rank sum test with continuity correction; alternative = two.sided	0.158	0.276
		AHAV 2	KW/MW Test	estimate = 2; statistic = 1500; p.value = 0.025; conf.low = 6.3e-05; conf.high = 3; method = Wilcoxon rank sum test with continuity correction; alternative = two.sided	0.0255	0.0594
		CMV IgG	KW/MW Test	estimate = -1; statistic = 180; p.value = 0.37; conf.low = -5; conf.high = 2; method = Wilcoxon rank sum test with continuity correction; alternative = two.sided	0.374	0.469

Impairment	Group	Feature	Method	Test output	p-value	p-value adjusted
		EBVEBNA IgG	KW/MW Test	estimate = -5.3e-05; statistic = 770; p.value = 0.66; conf.low = -2; conf.high = 1; method = Wilcoxon rank sum test with continuity correction; alternative = two.sided	0.658	0.658
		HBSAGII	KW/MW Test	estimate = -1; statistic = 280; p.value = 0.4; conf.low = -6; conf.high = 2; method = Wilcoxon rank sum test with continuity correction; alternative = two.sided	0.402	0.469
		HSV-1	KW/MW Test	estimate = -1; statistic = 1400; p.value = 0.017; conf.low = -3; conf.high = -5.6e-05; method = Wilcoxon rank sum test with continuity correction; alternative = two.sided	0.0168	0.0588
		HSV-2	KW/MW Test	estimate = -4; statistic = 240; p.value = 0.0063; conf.low = -7; conf.high = -1; method = Wilcoxon rank sum test with continuity correction; alternative = two.sided	0.00629	0.044
	Vitamins	B12 II	KW/MW Test	statistic = 1; p.value = 0.61; parameter = 2; method = Kruskal-Wallis rank sum test	0.606	0.606
		FOL III	KW/MW Test	statistic = 3; p.value = 0.23; parameter = 2; method = Kruskal-Wallis rank sum test	0.225	0.606
		VitD II	KW/MW Test	statistic = 1.2; p.value = 0.55; parameter = 2; method = Kruskal-Wallis rank sum test	0.552	0.606
MOCA abstraction	Demography	Age	Spearman correlation	estimate = -0.14; statistic = 790000; p.value = 0.078; method = Spearman's rank correlation rho; alternative = two.sided	0.0777	0.0777
		Sex	KW/MW Test	estimate = -3.9e-05; statistic = 2600; p.value = 0.045; conf.low = -3.9e-05; conf.high =	0.045	0.0777

Impairment	Group	Feature	Method	Test output	p-value	p-value adjusted
				5.2e-05; method = Wilcoxon rank sum test with continuity correction; alternative = two.sided		
	Depression	PHQ-9	Spearman correlation	estimate = -0.083; statistic = 750000; p.value = 0.3; method = Spearman's rank correlation rho; alternative = two.sided	0.295	0.295
	Genes	APP	GLM	df.residual = 130; residual.deviance = 18	0.907	0.907
		GRN	GLM	df.residual = 140; residual.deviance = 17	0.0171	0.0857
		MAPT	GLM	df.residual = 130; residual.deviance = 16	0.164	0.409
		PSEN1	GLM	df.residual = 140; residual.deviance = 18	0.549	0.907
		PSEN2	GLM	df.residual = 130; residual.deviance = 18	0.732	0.907
	Viruses	ACOV2 IgG	KW/MW Test	estimate = 0; statistic = 370; p.value = 0.37; conf.low = 0; conf.high = 0; method = Wilcoxon rank sum test with continuity correction; alternative = two.sided	0.372	0.521
		AHAV 2	KW/MW Test	estimate = 7.5e-05; statistic = 1200; p.value = 0.34; conf.low = -2.9e-05; conf.high = 5.5e-05; method = Wilcoxon rank sum test with continuity correction; alternative = two.sided	0.34	0.521
		CMV IgG	KW/MW Test	estimate = 0; statistic = 220; p.value = 0.52; conf.low = 0; conf.high = 0; method = Wilcoxon rank sum test with continuity correction; alternative = two.sided	0.517	0.603
		EBVEBNA IgG	KW/MW Test	estimate = 0; statistic = 730; p.value = 0.16; conf.low = 0; conf.high = 0; method =	0.162	0.378

Impairment	Group	Feature	Method	Test output	p-value	p-value adjusted
				Wilcoxon rank sum test with continuity correction; alternative = two.sided		
		HBSAGII	KW/MW Test	estimate = -2.2e-05; statistic = 240; p.value = 0.033; conf.low = -1; conf.high = -3.8e-05; method = Wilcoxon rank sum test with continuity correction; alternative = two.sided	0.0332	0.232
		HSV-1	KW/MW Test	estimate = -1.2e-05; statistic = 1700; p.value = 0.11; conf.low = -3.6e-05; conf.high = 8e-06; method = Wilcoxon rank sum test with continuity correction; alternative = two.sided	0.114	0.378
		HSV-2	KW/MW Test	estimate = -4.3e-06; statistic = 540; p.value = 1; conf.low = -7e-05; conf.high = 2.1e-05; method = Wilcoxon rank sum test with continuity correction; alternative = two.sided	1	1
	Vitamins	B12 II	KW/MW Test	statistic = 0.99; p.value = 0.61; parameter = 2; method = Kruskal-Wallis rank sum test	0.609	0.779
		FOL III	KW/MW Test	statistic = 0.5; p.value = 0.78; parameter = 2; method = Kruskal-Wallis rank sum test	0.779	0.779
		VitD II	KW/MW Test	statistic = 0.54; p.value = 0.76; parameter = 2; method = Kruskal-Wallis rank sum test	0.763	0.779
MOCA attention	Demography	Age	Spearman correlation	estimate = -0.13; statistic = 780000; p.value = 0.11; method = Spearman's rank correlation rho; alternative = two.sided	0.111	0.147
		Sex	KW/MW Test	estimate = -5.4e-05; statistic = 2600; p.value = 0.15; conf.low = -4.7e-05; conf.high = 1.9e-05; method = Wilcoxon rank sum test with continuity correction; alternative = two.sided	0.147	0.147

Impairment	Group	Feature	Method	Test output	p-value	p-value adjusted
	Depression	PHQ-9	Spearman correlation	estimate = -0.082; statistic = 750000; p.value = 0.3; method = Spearman's rank correlation rho; alternative = two.sided	0.299	0.299
	Genes	APP	GLM	df.residual = 130; residual.deviance = 140	0.366	0.489
		GRN	GLM	df.residual = 140; residual.deviance = 150	0.457	0.489
		MAPT	GLM	df.residual = 130; residual.deviance = 140	0.442	0.489
		PSEN1	GLM	df.residual = 140; residual.deviance = 150	0.489	0.489
		PSEN2	GLM	df.residual = 130; residual.deviance = 140	0.471	0.489
	Viruses	ACOV2 IgG	KW/MW Test	estimate = -3.5e-05; statistic = 370; p.value = 0.58; conf.low = -1; conf.high = 7.5e-06; method = Wilcoxon rank sum test with continuity correction; alternative = two.sided	0.579	0.579
		AHAV 2	KW/MW Test	estimate = 3.3e-05; statistic = 1400; p.value = 0.14; conf.low = -4.2e-05; conf.high = 1.6e-05; method = Wilcoxon rank sum test with continuity correction; alternative = two.sided	0.137	0.24
		CMV IgG	KW/MW Test	estimate = -5.7e-05; statistic = 130; p.value = 0.085; conf.low = -1; conf.high = 0; method = Wilcoxon rank sum test with continuity correction; alternative = two.sided	0.0849	0.198
		EBVEBNA IgG	KW/MW Test	estimate = -3.6e-05; statistic = 600; p.value = 0.051; conf.low = -1; conf.high = 8.4e-06; method = Wilcoxon rank sum test with continuity correction; alternative = two.sided	0.0513	0.18

Impairment	Group	Feature	Method	Test output	p-value	p-value adjusted
		HBSAGII	KW/MW Test	estimate = -6.8e-06; statistic = 250; p.value = 0.21; conf.low = -2; conf.high = 2.5e-05; method = Wilcoxon rank sum test with continuity correction; alternative = two.sided	0.208	0.243
		HSV-1	KW/MW Test	estimate = -6.5e-05; statistic = 1600; p.value = 0.21; conf.low = -3.8e-05; conf.high = 4.8e-05; method = Wilcoxon rank sum test with continuity correction; alternative = two.sided	0.208	0.243
		HSV-2	KW/MW Test	estimate = -1; statistic = 320; p.value = 0.019; conf.low = -2; conf.high = -6.2e-07; method = Wilcoxon rank sum test with continuity correction; alternative = two.sided	0.0188	0.131
	Vitamins	B12 II	KW/MW Test	statistic = 0.57; p.value = 0.75; parameter = 2; method = Kruskal-Wallis rank sum test	0.751	0.751
		FOL III	KW/MW Test	statistic = 7.5; p.value = 0.023; parameter = 2; method = Kruskal-Wallis rank sum test	0.0234	0.0702
		VitD II	KW/MW Test	statistic = 3.3; p.value = 0.19; parameter = 2; method = Kruskal-Wallis rank sum test	0.191	0.286
MOCA language	Demography	Age	Spearman correlation	estimate = -0.18; statistic = 820000; p.value = 0.026; method = Spearman's rank correlation rho; alternative = two.sided	0.0262	0.0289
		Sex	KW/MW Test	estimate = -5.6e-05; statistic = 2400; p.value = 0.029; conf.low = -1; conf.high = -5.9e-06; method = Wilcoxon rank sum test with continuity correction; alternative = two.sided	0.0289	0.0289

Impairment	Group	Feature	Method	Test output	p-value	p-value adjusted
	Depression	PHQ-9	Spearman correlation	estimate = -0.19; statistic = 830000; p.value = 0.016; method = Spearman's rank correlation rho; alternative = two.sided	0.016	0.016
	Genes	APP	GLM	df.residual = 130; residual.deviance = 80	0.682	0.682
		GRN	GLM	df.residual = 140; residual.deviance = 80	0.0705	0.129
		MAPT	GLM	df.residual = 130; residual.deviance = 70	0.0417	0.129
		PSEN1	GLM	df.residual = 140; residual.deviance = 82	0.547	0.682
		PSEN2	GLM	df.residual = 130; residual.deviance = 75	0.0772	0.129
	Viruses	ACOV2 IgG	KW/MW Test	estimate = 7.6e-06; statistic = 450; p.value = 0.76; conf.low = -1; conf.high = 1; method = Wilcoxon rank sum test with continuity correction; alternative = two.sided	0.758	0.908
		AHAV 2	KW/MW Test	estimate = 3.1e-05; statistic = 1300; p.value = 0.44; conf.low = -2.1e-05; conf.high = 7.9e-05; method = Wilcoxon rank sum test with continuity correction; alternative = two.sided	0.444	0.908
		CMV IgG	KW/MW Test	estimate = -4e-05; statistic = 230; p.value = 0.91; conf.low = -1; conf.high = 1; method = Wilcoxon rank sum test with continuity correction; alternative = two.sided	0.908	0.908
		EBVEBNA IgG	KW/MW Test	estimate = 2.1e-05; statistic = 860; p.value = 0.85; conf.low = -1.7e-05; conf.high = 6.7e-05; method = Wilcoxon rank sum test with continuity correction; alternative = two.sided	0.852	0.908

Impairment	Group	Feature	Method	Test output	p-value	p-value adjusted
		HBSAGII	KW/MW Test	estimate = 4.5e-05; statistic = 390; p.value = 0.66; conf.low = -1; conf.high = 1; method = Wilcoxon rank sum test with continuity correction; alternative = two.sided	0.662	0.908
		HSV-1	KW/MW Test	estimate = -8.4e-05; statistic = 1700; p.value = 0.49; conf.low = -2.8e-05; conf.high = 1.8e-05; method = Wilcoxon rank sum test with continuity correction; alternative = two.sided	0.494	0.908
		HSV-2	KW/MW Test	estimate = -2.9e-05; statistic = 390; p.value = 0.14; conf.low = -1; conf.high = 2.5e-05; method = Wilcoxon rank sum test with continuity correction; alternative = two.sided	0.143	0.908
	Vitamins	B12 II	KW/MW Test	statistic = 2.1; p.value = 0.35; parameter = 2; method = Kruskal-Wallis rank sum test	0.351	0.699
		FOL III	KW/MW Test	statistic = 0.51; p.value = 0.78; parameter = 2; method = Kruskal-Wallis rank sum test	0.776	0.776
		VitD II	KW/MW Test	statistic = 1.5; p.value = 0.47; parameter = 2; method = Kruskal-Wallis rank sum test	0.466	0.699
MOCA naming	Demography	Age	Spearman correlation	estimate = -0.11; statistic = 770000; p.value = 0.16; method = Spearman's rank correlation rho; alternative = two.sided	0.157	0.157
		Sex	KW/MW Test	estimate = -1.6e-05; statistic = 2600; p.value = 0.044; conf.low = -8.8e-05; conf.high = 6.3e-05; method = Wilcoxon rank sum test with continuity correction; alternative = two.sided	0.0443	0.0886

Impairment	Group	Feature	Method	Test output	p-value	p-value adjusted
	Depression	PHQ-9	Spearman correlation	estimate = -0.069; statistic = 740000; p.value = 0.39; method = Spearman's rank correlation rho; alternative = two.sided	0.386	0.386
	Genes	APP	GLM	df.residual = 130; residual.deviance = 22	0.882	0.944
		GRN	GLM	df.residual = 140; residual.deviance = 22	0.235	0.485
		MAPT	GLM	df.residual = 130; residual.deviance = 19	0.0885	0.443
		PSEN1	GLM	df.residual = 140; residual.deviance = 22	0.291	0.485
		PSEN2	GLM	df.residual = 130; residual.deviance = 23	0.944	0.944
	Viruses	ACOV2 IgG	KW/MW Test	estimate = 8e-05; statistic = 440; p.value = 0.85; conf.low = -9.8e-06; conf.high = 6.3e-06; method = Wilcoxon rank sum test with continuity correction; alternative = two.sided	0.85	0.976
		AHAV 2	KW/MW Test	estimate = 6.2e-05; statistic = 1200; p.value = 0.51; conf.low = -3.7e-05; conf.high = 1.8e-05; method = Wilcoxon rank sum test with continuity correction; alternative = two.sided	0.509	0.713
		CMV IgG	KW/MW Test	estimate = 0; statistic = 210; p.value = 0.43; conf.low = -7e-05; conf.high = 0; method = Wilcoxon rank sum test with continuity correction; alternative = two.sided	0.427	0.713
		EBVEBNA IgG	KW/MW Test	estimate = -4.8e-06; statistic = 830; p.value = 0.98; conf.low = -1.8e-05; conf.high = 5.7e-05; method = Wilcoxon rank sum test with continuity correction; alternative = two.sided	0.976	0.976

Impairment	Group	Feature	Method	Test output	p-value	p-value adjusted
		HBSAGII	KW/MW Test	estimate = -1; statistic = 170; p.value = 0.00086; conf.low = -2; conf.high = -8.5e-05; method = Wilcoxon rank sum test with continuity correction; alternative = two.sided	0.000857	0.006
		HSV-1	KW/MW Test	estimate = -3.5e-05; statistic = 1600; p.value = 0.03; conf.low = -1.6e-05; conf.high = 2.5e-05; method = Wilcoxon rank sum test with continuity correction; alternative = two.sided	0.0297	0.0693
		HSV-2	KW/MW Test	estimate = -5.8e-05; statistic = 410; p.value = 0.029; conf.low = -1.4e-05; conf.high = 4.9e-05; method = Wilcoxon rank sum test with continuity correction; alternative = two.sided	0.0286	0.0693
	Vitamins	B12 II	KW/MW Test	statistic = 0.51; p.value = 0.77; parameter = 2; method = Kruskal-Wallis rank sum test	0.774	0.841
		FOL III	KW/MW Test	statistic = 2.1; p.value = 0.35; parameter = 2; method = Kruskal-Wallis rank sum test	0.35	0.841
		VitD II	KW/MW Test	statistic = 0.35; p.value = 0.84; parameter = 2; method = Kruskal-Wallis rank sum test	0.841	0.841
MOCA orientation	Demography	Age	Spearman correlation	estimate = -0.048; statistic = 730000; p.value = 0.54; method = Spearman's rank correlation rho; alternative = two.sided	0.542	0.542
		Sex	KW/MW Test	estimate = -3.9e-05; statistic = 2800; p.value = 0.31; conf.low = -6.8e-05; conf.high = 6.3e-05; method = Wilcoxon rank sum test with continuity correction; alternative = two.sided	0.312	0.542

Impairment	Group	Feature	Method	Test output	p-value	p-value adjusted
	Depression	PHQ-9	Spearman correlation	estimate = -0.16; statistic = 8e+05; p.value = 0.048; method = Spearman's rank correlation rho; alternative = two.sided	0.0475	0.0475
	Genes	APP	GLM	df.residual = 130; residual.deviance = 17	0.641	0.944
		GRN	GLM	df.residual = 140; residual.deviance = 18	0.604	0.944
		MAPT	GLM	df.residual = 130; residual.deviance = 17	0.787	0.944
		PSEN1	GLM	df.residual = 140; residual.deviance = 18	0.936	0.944
		PSEN2	GLM	df.residual = 130; residual.deviance = 18	0.944	0.944
	Viruses	ACOV2 IgG	KW/MW Test	estimate = 2.6e-05; statistic = 460; p.value = 0.53; conf.low = -1.8e-05; conf.high = 4.5e-05; method = Wilcoxon rank sum test with continuity correction; alternative = two.sided	0.526	0.736
		AHAV 2	KW/MW Test	estimate = 5.1e-05; statistic = 1200; p.value = 0.84; conf.low = -1e-05; conf.high = 4.6e-05; method = Wilcoxon rank sum test with continuity correction; alternative = two.sided	0.842	0.842
		CMV IgG	KW/MW Test	estimate = 0; statistic = 210; p.value = 0.5; conf.low = -5.3e-05; conf.high = 0; method = Wilcoxon rank sum test with continuity correction; alternative = two.sided	0.497	0.736
		EBVEBNA IgG	KW/MW Test	estimate = 2.5e-05; statistic = 850; p.value = 0.82; conf.low = -2.2e-05; conf.high = 1.8e-05; method = Wilcoxon rank sum test with continuity correction; alternative = two.sided	0.82	0.842

Impairment	Group	Feature	Method	Test output	p-value	p-value adjusted
		HBSAGII	KW/MW Test	estimate = 0; statistic = 400; p.value = 0.43; conf.low = 0; conf.high = 0; method = Wilcoxon rank sum test with continuity correction; alternative = two.sided	0.434	0.736
		HSV-1	KW/MW Test	estimate = 0; statistic = 1600; p.value = 0.015; conf.low = 0; conf.high = 0; method = Wilcoxon rank sum test with continuity correction; alternative = two.sided	0.0155	0.108
		HSV-2	KW/MW Test	estimate = -1.8e-05; statistic = 450; p.value = 0.099; conf.low = -5.4e-05; conf.high = 7.6e-05; method = Wilcoxon rank sum test with continuity correction; alternative = two.sided	0.099	0.347
	Vitamins	B12 II	KW/MW Test	statistic = 2.2; p.value = 0.34; parameter = 2; method = Kruskal-Wallis rank sum test	0.336	0.765
		FOL III	KW/MW Test	statistic = 0.53; p.value = 0.77; parameter = 2; method = Kruskal-Wallis rank sum test	0.765	0.765
		VitD II	KW/MW Test	statistic = 1.3; p.value = 0.53; parameter = 2; method = Kruskal-Wallis rank sum test	0.533	0.765
MOCA visuospecial/executive	Demography	Age	Spearman correlation	estimate = -0.083; statistic = 750000; p.value = 0.29; method = Spearman's rank correlation rho; alternative = two.sided	0.293	0.293
		Sex	KW/MW Test	estimate = -3.8e-05; statistic = 2400; p.value = 0.022; conf.low = -3.3e-05; conf.high = -6.3e-05; method = Wilcoxon rank sum test with continuity correction; alternative = two.sided	0.0223	0.0445

Impairment	Group	Feature	Method	Test output	p-value	p-value adjusted
	Depression	PHQ-9	Spearman correlation	estimate = -0.056; statistic = 730000; p.value = 0.48; method = Spearman's rank correlation rho; alternative = two.sided	0.483	0.483
	Genes	APP	GLM	df.residual = 130; residual.deviance = 95	0.935	0.935
		GRN	GLM	df.residual = 140; residual.deviance = 92	0.0538	0.269
		MAPT	GLM	df.residual = 130; residual.deviance = 84	0.108	0.271
		PSEN1	GLM	df.residual = 140; residual.deviance = 93	0.425	0.709
		PSEN2	GLM	df.residual = 130; residual.deviance = 94	0.75	0.935
	Viruses	ACOV2 IgG	KW/MW Test	estimate = 1; statistic = 570; p.value = 0.1; conf.low = -3.8e-05; conf.high = 1; method = Wilcoxon rank sum test with continuity correction; alternative = two.sided	0.102	0.15
		AHAV 2	KW/MW Test	estimate = 4.5e-06; statistic = 1500; p.value = 0.024; conf.low = 1.2e-05; conf.high = 1; method = Wilcoxon rank sum test with continuity correction; alternative = two.sided	0.024	0.0701
		CMV IgG	KW/MW Test	estimate = -8e-05; statistic = 140; p.value = 0.11; conf.low = -1; conf.high = 0; method = Wilcoxon rank sum test with continuity correction; alternative = two.sided	0.107	0.15
		EBVEBNA IgG	KW/MW Test	estimate = -2e-05; statistic = 700; p.value = 0.28; conf.low = -1.6e-05; conf.high = 7e-05; method = Wilcoxon rank sum test with continuity correction; alternative = two.sided	0.281	0.328

Impairment	Group	Feature	Method	Test output	p-value	p-value adjusted
		HBSAGII	KW/MW Test	estimate = -5.6e-05; statistic = 320; p.value = 0.67; conf.low = -1; conf.high = 1.9e-05; method = Wilcoxon rank sum test with continuity correction; alternative = two.sided	0.669	0.669
		HSV-1	KW/MW Test	estimate = -4.2e-05; statistic = 1400; p.value = 0.011; conf.low = -1.8e-05; conf.high = -5.1e-05; method = Wilcoxon rank sum test with continuity correction; alternative = two.sided	0.0109	0.0701
		HSV-2	KW/MW Test	estimate = -1; statistic = 330; p.value = 0.03; conf.low = -1; conf.high = -2e-05; method = Wilcoxon rank sum test with continuity correction; alternative = two.sided	0.03	0.0701
	Vitamins	B12 II	KW/MW Test	statistic = 0.39; p.value = 0.82; parameter = 2; method = Kruskal-Wallis rank sum test	0.823	0.823
		FOL III	KW/MW Test	statistic = 0.43; p.value = 0.81; parameter = 2; method = Kruskal-Wallis rank sum test	0.807	0.823
		VitD II	KW/MW Test	statistic = 1.7; p.value = 0.42; parameter = 2; method = Kruskal-Wallis rank sum test	0.424	0.823
Memory impairment after COVID-19	Demography	Age	KW/MW Test	statistic = 0.52; p.value = 0.47; parameter = 1; method = Kruskal-Wallis rank sum test	0.47	0.47
		Sex	Chi-squared test	statistic = 2.1; p.value = 0.15; parameter = 1; method = Pearson's Chi-squared test with Yates' continuity correction	0.151	0.303
	Depression	PHQ-9	KW/MW Test	statistic = 14; p.value = 0.00022; parameter = 1; method = Kruskal-Wallis rank sum test	0.000221	0.000221

Impairment	Group	Feature	Method	Test output	p-value	p-value adjusted
	Genes	APP	GLM	df.residual = 130; residual.deviance = 190	0.495	0.781
		GRN	GLM	df.residual = 140; residual.deviance = 200	0.368	0.781
		MAPT	GLM	df.residual = 130; residual.deviance = 190	0.761	0.781
		PSEN1	GLM	df.residual = 140; residual.deviance = 200	0.704	0.781
		PSEN2	GLM	df.residual = 130; residual.deviance = 190	0.781	0.781
	Viruses	ACOV2 IgG	Chi-squared test	statistic = 2.1e-30; p.value = 1; parameter = 1; method = Pearson's Chi-squared test with Yates' continuity correction	1	1
		AHAV 2	Chi-squared test	statistic = 1.3; p.value = 0.25; parameter = 1; method = Pearson's Chi-squared test with Yates' continuity correction	0.252	0.961
		CMV IgG	Chi-squared test	statistic = 0.071; p.value = 0.79; parameter = 1; method = Pearson's Chi-squared test with Yates' continuity correction	0.79	1
		EBVEBNA IgG	Chi-squared test	statistic = 0.67; p.value = 0.41; parameter = 1; method = Pearson's Chi-squared test with Yates' continuity correction	0.412	0.961
		HBSAGII	Chi-squared test	statistic = 0.061; p.value = 0.8; parameter = 1; method = Pearson's Chi-squared test with Yates' continuity correction	0.805	1
		HSV-1	Chi-squared test	statistic = 0.9; p.value = 0.34; parameter = 1; method = Pearson's Chi-squared test with Yates' continuity correction	0.342	0.961

Impairment	Group	Feature	Method	Test output	p-value	p-value adjusted
		HSV-2	Chi-squared test	statistic = 1.4e-29; p.value = 1; parameter = 1; method = Pearson's Chi-squared test with Yates' continuity correction	1	1
	Vitamins	B12 II	Chi-squared test	statistic = 2.1; p.value = 0.35; parameter = 2; method = Pearson's Chi-squared test	0.347	0.347
		FOL III	Chi-squared test	statistic = 3.3; p.value = 0.2; parameter = 2; method = Pearson's Chi-squared test	0.197	0.347
		VitD II	Chi-squared test	statistic = 2.5; p.value = 0.29; parameter = 2; method = Pearson's Chi-squared test	0.293	0.347
RBANS Attention	Demography	Age	Spearman correlation	estimate = -0.079; statistic = 760000; p.value = 0.32; method = Spearman's rank correlation rho; alternative = two.sided	0.317	0.401
		Sex	KW/MW Test	estimate = -3; statistic = 2700; p.value = 0.4; conf.low = -7; conf.high = 3; method = Wilcoxon rank sum test with continuity correction; alternative = two.sided	0.401	0.401
	Depression	PHQ-9	Spearman correlation	estimate = -0.21; statistic = 840000; p.value = 0.0079; method = Spearman's rank correlation rho; alternative = two.sided	0.00788	0.00788
	Genes	APP	GLM	df.residual = 130; residual.deviance = 34000	0.571	0.588
		GRN	GLM	df.residual = 140; residual.deviance = 34000	0.0403	0.202
		MAPT	GLM	df.residual = 130; residual.deviance = 33000	0.433	0.588
		PSEN1	GLM	df.residual = 140; residual.deviance = 35000	0.583	0.588
		PSEN2	GLM	df.residual = 130; residual.deviance = 35000	0.588	0.588

Impairment	Group	Feature	Method	Test output	p-value	p-value adjusted
	Viruses	ACOV2 IgG	KW/MW Test	estimate = 12; statistic = 610; p.value = 0.076; conf.low = -3; conf.high = 26; method = Wilcoxon rank sum test with continuity correction; alternative = two.sided	0.0762	0.267
		AHAV 2	KW/MW Test	estimate = 6; statistic = 1400; p.value = 0.15; conf.low = -3; conf.high = 13; method = Wilcoxon rank sum test with continuity correction; alternative = two.sided	0.148	0.345
		CMV IgG	KW/MW Test	estimate = -8.9; statistic = 190; p.value = 0.43; conf.low = -27; conf.high = 12; method = Wilcoxon rank sum test with continuity correction; alternative = two.sided	0.431	0.434
		EBVEBNA IgG	KW/MW Test	estimate = 4; statistic = 970; p.value = 0.36; conf.low = -4; conf.high = 12; method = Wilcoxon rank sum test with continuity correction; alternative = two.sided	0.358	0.434
		HBSAGII	KW/MW Test	estimate = -6; statistic = 280; p.value = 0.43; conf.low = -21; conf.high = 7; method = Wilcoxon rank sum test with continuity correction; alternative = two.sided	0.434	0.434
		HSV-1	KW/MW Test	estimate = -3; statistic = 1700; p.value = 0.29; conf.low = -9; conf.high = 3; method = Wilcoxon rank sum test with continuity correction; alternative = two.sided	0.285	0.434
		HSV-2	KW/MW Test	estimate = -13; statistic = 260; p.value = 0.0098; conf.low = -20; conf.high = -3; method = Wilcoxon rank sum test with continuity correction; alternative = two.sided	0.00984	0.0689

Impairment	Group	Feature	Method	Test output	p-value	p-value adjusted
	Vitamins	B12 II	KW/MW Test	statistic = 0.4; p.value = 0.82; parameter = 2; method = Kruskal-Wallis rank sum test	0.818	0.818
		FOL III	KW/MW Test	statistic = 1.6; p.value = 0.45; parameter = 2; method = Kruskal-Wallis rank sum test	0.446	0.669
		VitD II	KW/MW Test	statistic = 3.1; p.value = 0.22; parameter = 2; method = Kruskal-Wallis rank sum test	0.216	0.648
RBANS Delayed Memory	Demography	Age	Spearman correlation	estimate = -0.074; statistic = 760000; p.value = 0.35; method = Spearman's rank correlation rho; alternative = two.sided	0.35	0.661
		Sex	KW/MW Test	estimate = 1; statistic = 3100; p.value = 0.66; conf.low = -4; conf.high = 6; method = Wilcoxon rank sum test with continuity correction; alternative = two.sided	0.661	0.661
	Depression	PHQ-9	Spearman correlation	estimate = -0.076; statistic = 750000; p.value = 0.34; method = Spearman's rank correlation rho; alternative = two.sided	0.341	0.341
	Genes	APP	GLM	df.residual = 130; residual.deviance = 33000	0.13	0.326
		GRN	GLM	df.residual = 140; residual.deviance = 37000	0.25	0.417
		MAPT	GLM	df.residual = 130; residual.deviance = 35000	0.646	0.646
		PSEN1	GLM	df.residual = 140; residual.deviance = 34000	0.0553	0.276
		PSEN2	GLM	df.residual = 130; residual.deviance = 35000	0.47	0.587
	Viruses	ACOV2 IgG	KW/MW Test	estimate = 8; statistic = 600; p.value = 0.1; conf.low = -3; conf.high = 22; method =	0.0999	0.233

Impairment	Group	Feature	Method	Test output	p-value	p-value adjusted
				Wilcoxon rank sum test with continuity correction; alternative = two.sided		
		AHAV 2	KW/MW Test	estimate = 3; statistic = 1400; p.value = 0.27; conf.low = -3; conf.high = 11; method = Wilcoxon rank sum test with continuity correction; alternative = two.sided	0.27	0.315
		CMV IgG	KW/MW Test	estimate = -11; statistic = 160; p.value = 0.26; conf.low = -27; conf.high = 24; method = Wilcoxon rank sum test with continuity correction; alternative = two.sided	0.261	0.315
		EBVEBNA IgG	KW/MW Test	estimate = -2; statistic = 760; p.value = 0.58; conf.low = -10; conf.high = 6; method = Wilcoxon rank sum test with continuity correction; alternative = two.sided	0.58	0.58
		HBSAGII	KW/MW Test	estimate = -8; statistic = 220; p.value = 0.15; conf.low = -20; conf.high = 3; method = Wilcoxon rank sum test with continuity correction; alternative = two.sided	0.151	0.265
		HSV-1	KW/MW Test	estimate = -8; statistic = 1400; p.value = 0.0087; conf.low = -13; conf.high = -2; method = Wilcoxon rank sum test with continuity correction; alternative = two.sided	0.00874	0.0612
		HSV-2	KW/MW Test	estimate = -8; statistic = 340; p.value = 0.06; conf.low = -17; conf.high = 9.5e-06; method = Wilcoxon rank sum test with continuity correction; alternative = two.sided	0.0601	0.21
	Vitamins	B12 II	KW/MW Test	statistic = 0.34; p.value = 0.84; parameter = 2; method = Kruskal-Wallis rank sum test	0.842	0.842

Impairment	Group	Feature	Method	Test output	p-value	p-value adjusted
		FOL III	KW/MW Test	statistic = 0.38; p.value = 0.83; parameter = 2; method = Kruskal-Wallis rank sum test	0.828	0.842
		VitD II	KW/MW Test	statistic = 1.1; p.value = 0.58; parameter = 2; method = Kruskal-Wallis rank sum test	0.578	0.842
RBANS Immediate Memory	Demography	Age	Spearman correlation	estimate = -0.076; statistic = 760000; p.value = 0.34; method = Spearman's rank correlation rho; alternative = two.sided	0.339	0.679
		Sex	KW/MW Test	estimate = 2.4e-05; statistic = 3100; p.value = 0.77; conf.low = -5; conf.high = 6; method = Wilcoxon rank sum test with continuity correction; alternative = two.sided	0.772	0.772
	Depression	PHQ-9	Spearman correlation	estimate = -0.21; statistic = 840000; p.value = 0.0072; method = Spearman's rank correlation rho; alternative = two.sided	0.00722	0.00722
	Genes	APP	GLM	df.residual = 130; residual.deviance = 36000	0.0646	0.162
		GRN	GLM	df.residual = 140; residual.deviance = 39000	0.0251	0.125
		MAPT	GLM	df.residual = 130; residual.deviance = 39000	0.733	0.733
		PSEN1	GLM	df.residual = 140; residual.deviance = 40000	0.28	0.411
		PSEN2	GLM	df.residual = 130; residual.deviance = 39000	0.329	0.411
	Viruses	ACOV2 IgG	KW/MW Test	estimate = 3; statistic = 470; p.value = 0.7; conf.low = -13; conf.high = 19; method = Wilcoxon rank sum test with continuity correction; alternative = two.sided	0.704	0.839

Impairment	Group	Feature	Method	Test output	p-value	p-value adjusted
		AHAV 2	KW/MW Test	estimate = 3; statistic = 1300; p.value = 0.44; conf.low = -5; conf.high = 11; method = Wilcoxon rank sum test with continuity correction; alternative = two.sided	0.442	0.839
		CMV IgG	KW/MW Test	estimate = -3; statistic = 220; p.value = 0.74; conf.low = -24; conf.high = 18; method = Wilcoxon rank sum test with continuity correction; alternative = two.sided	0.741	0.839
		EBVEBNA IgG	KW/MW Test	estimate = -3; statistic = 770; p.value = 0.62; conf.low = -13; conf.high = 7; method = Wilcoxon rank sum test with continuity correction; alternative = two.sided	0.623	0.839
		HBSAGII	KW/MW Test	estimate = -21; statistic = 120; p.value = 0.011; conf.low = -35; conf.high = -5; method = Wilcoxon rank sum test with continuity correction; alternative = two.sided	0.0107	0.0751
		HSV-1	KW/MW Test	estimate = -8; statistic = 1400; p.value = 0.024; conf.low = -14; conf.high = -1.6e-05; method = Wilcoxon rank sum test with continuity correction; alternative = two.sided	0.0238	0.0832
		HSV-2	KW/MW Test	estimate = -3.6e-05; statistic = 520; p.value = 0.84; conf.low = -13; conf.high = 9; method = Wilcoxon rank sum test with continuity correction; alternative = two.sided	0.839	0.839
	Vitamins	B12 II	KW/MW Test	statistic = 1; p.value = 0.6; parameter = 2; method = Kruskal-Wallis rank sum test	0.605	0.791
		FOL III	KW/MW Test	statistic = 0.85; p.value = 0.65; parameter = 2; method = Kruskal-Wallis rank sum test	0.652	0.791

Impairment	Group	Feature	Method	Test output	p-value	p-value adjusted
		VitD II	KW/MW Test	statistic = 0.47; p.value = 0.79; parameter = 2; method = Kruskal-Wallis rank sum test	0.791	0.791
RBANS Language	Demography	Age	Spearman correlation	estimate = 0.13; statistic = 610000; p.value = 0.091; method = Spearman's rank correlation rho; alternative = two.sided	0.0906	0.181
		Sex	KW/MW Test	estimate = -3; statistic = 2600; p.value = 0.24; conf.low = -8; conf.high = 2; method = Wilcoxon rank sum test with continuity correction; alternative = two.sided	0.241	0.241
	Depression	PHQ-9	Spearman correlation	estimate = -0.011; statistic = 7e+05; p.value = 0.89; method = Spearman's rank correlation rho; alternative = two.sided	0.891	0.891
	Genes	APP	GLM	df.residual = 130; residual.deviance = 38000	0.803	0.846
		GRN	GLM	df.residual = 140; residual.deviance = 39000	0.25	0.708
		MAPT	GLM	df.residual = 130; residual.deviance = 37000	0.575	0.846
		PSEN1	GLM	df.residual = 140; residual.deviance = 40000	0.846	0.846
		PSEN2	GLM	df.residual = 130; residual.deviance = 37000	0.283	0.708
	Viruses	ACOV2 IgG	KW/MW Test	estimate = 9; statistic = 570; p.value = 0.17; conf.low = -4; conf.high = 19; method = Wilcoxon rank sum test with continuity correction; alternative = two.sided	0.167	0.285
		AHAV 2	KW/MW Test	estimate = -6; statistic = 910; p.value = 0.096; conf.low = -13; conf.high = 1; method = Wilcoxon rank sum test with continuity correction; alternative = two.sided	0.0958	0.285

Impairment	Group	Feature	Method	Test output	p-value	p-value adjusted
		CMV IgG	KW/MW Test	estimate = 5; statistic = 280; p.value = 0.56; conf.low = -14; conf.high = 18; method = Wilcoxon rank sum test with continuity correction; alternative = two.sided	0.56	0.653
		EBVEBNA IgG	KW/MW Test	estimate = 8; statistic = 1000; p.value = 0.2; conf.low = -4; conf.high = 18; method = Wilcoxon rank sum test with continuity correction; alternative = two.sided	0.204	0.285
		HBSAGII	KW/MW Test	estimate = -14; statistic = 220; p.value = 0.15; conf.low = -39; conf.high = 6; method = Wilcoxon rank sum test with continuity correction; alternative = two.sided	0.151	0.285
		HSV-1	KW/MW Test	estimate = -4; statistic = 1600; p.value = 0.15; conf.low = -9; conf.high = 1; method = Wilcoxon rank sum test with continuity correction; alternative = two.sided	0.153	0.285
		HSV-2	KW/MW Test	estimate = -2; statistic = 500; p.value = 0.73; conf.low = -12; conf.high = 7; method = Wilcoxon rank sum test with continuity correction; alternative = two.sided	0.732	0.732
	Vitamins	B12 II	KW/MW Test	statistic = 0.72; p.value = 0.7; parameter = 2; method = Kruskal-Wallis rank sum test	0.699	0.699
		FOL III	KW/MW Test	statistic = 2.1; p.value = 0.35; parameter = 2; method = Kruskal-Wallis rank sum test	0.354	0.699
		VitD II	KW/MW Test	statistic = 1; p.value = 0.6; parameter = 2; method = Kruskal-Wallis rank sum test	0.599	0.699

Impairment	Group	Feature	Method	Test output	p-value	p-value adjusted
RBANS Total Scale of Index Scores	Demography	Age	Spearman correlation	estimate = -0.061; statistic = 750000; p.value = 0.44; method = Spearman's rank correlation rho; alternative = two.sided	0.444	0.444
		Sex	KW/MW Test	estimate = -3; statistic = 2600; p.value = 0.17; conf.low = -7; conf.high = 1; method = Wilcoxon rank sum test with continuity correction; alternative = two.sided	0.169	0.339
	Depression	PHQ-9	Spearman correlation	estimate = -0.22; statistic = 850000; p.value = 0.0054; method = Spearman's rank correlation rho; alternative = two.sided	0.0054	0.0054
	Genes	APP	GLM	df.residual = 130; residual.deviance = 24000	0.347	0.433
		GRN	GLM	df.residual = 140; residual.deviance = 25000	0.0329	0.165
		MAPT	GLM	df.residual = 130; residual.deviance = 24000	0.325	0.433
		PSEN1	GLM	df.residual = 140; residual.deviance = 26000	0.459	0.459
		PSEN2	GLM	df.residual = 130; residual.deviance = 24000	0.169	0.424
	Viruses	ACOV2 IgG	KW/MW Test	estimate = 9; statistic = 610; p.value = 0.078; conf.low = -1; conf.high = 19; method = Wilcoxon rank sum test with continuity correction; alternative = two.sided	0.0783	0.155
		AHAV 2	KW/MW Test	estimate = 5; statistic = 1400; p.value = 0.13; conf.low = -2; conf.high = 11; method = Wilcoxon rank sum test with continuity correction; alternative = two.sided	0.127	0.178
		CMV IgG	KW/MW Test	estimate = -7; statistic = 200; p.value = 0.57; conf.low = -28; conf.high = 14; method =	0.57	0.665

Impairment	Group	Feature	Method	Test output	p-value	p-value adjusted
				Wilcoxon rank sum test with continuity correction; alternative = two.sided		
		EBVEBNA IgG	KW/MW Test	estimate = -1; statistic = 820; p.value = 0.88; conf.low = -8; conf.high = 7; method = Wilcoxon rank sum test with continuity correction; alternative = two.sided	0.881	0.881
		HBSAGII	KW/MW Test	estimate = -10; statistic = 200; p.value = 0.088; conf.low = -24; conf.high = 2; method = Wilcoxon rank sum test with continuity correction; alternative = two.sided	0.0884	0.155
		HSV-1	KW/MW Test	estimate = -8; statistic = 1300; p.value = 0.0028; conf.low = -13; conf.high = -3; method = Wilcoxon rank sum test with continuity correction; alternative = two.sided	0.00285	0.0199
		HSV-2	KW/MW Test	estimate = -9; statistic = 330; p.value = 0.052; conf.low = -18; conf.high = 3.2e-05; method = Wilcoxon rank sum test with continuity correction; alternative = two.sided	0.0521	0.155
	Vitamins	B12 II	KW/MW Test	statistic = 0.59; p.value = 0.75; parameter = 2; method = Kruskal-Wallis rank sum test	0.746	0.96
		FOL III	KW/MW Test	statistic = 0.081; p.value = 0.96; parameter = 2; method = Kruskal-Wallis rank sum test	0.96	0.96
		VitD II	KW/MW Test	statistic = 0.61; p.value = 0.74; parameter = 2; method = Kruskal-Wallis rank sum test	0.736	0.96
RBANS Visuospatial Constructional	Demography	Age	Spearman correlation	estimate = -0.11; statistic = 780000; p.value = 0.18; method = Spearman's rank correlation rho; alternative = two.sided	0.178	0.178

Impairment	Group	Feature	Method	Test output	p-value	p-value adjusted
		Sex	KW/MW Test	estimate = -9; statistic = 2000; p.value = 0.00046; conf.low = -13; conf.high = -4; method = Wilcoxon rank sum test with continuity correction; alternative = two.sided	0.000457	0.000915
	Depression	PHQ-9	Spearman correlation	estimate = -0.13; statistic = 780000; p.value = 0.11; method = Spearman's rank correlation rho; alternative = two.sided	0.108	0.108
	Genes	APP	GLM	df.residual = 130; residual.deviance = 33000	0.312	0.508
		GRN	GLM	df.residual = 140; residual.deviance = 34000	0.0338	0.169
		MAPT	GLM	df.residual = 130; residual.deviance = 33000	0.317	0.508
		PSEN1	GLM	df.residual = 140; residual.deviance = 35000	0.406	0.508
		PSEN2	GLM	df.residual = 130; residual.deviance = 35000	0.744	0.744
	Viruses	ACOV2 IgG	KW/MW Test	estimate = 3; statistic = 480; p.value = 0.61; conf.low = -10; conf.high = 16; method = Wilcoxon rank sum test with continuity correction; alternative = two.sided	0.615	0.658
		AHAV 2	KW/MW Test	estimate = 10; statistic = 1700; p.value = 0.0025; conf.low = 3; conf.high = 17; method = Wilcoxon rank sum test with continuity correction; alternative = two.sided	0.00247	0.0173
		CMV IgG	KW/MW Test	estimate = -5; statistic = 180; p.value = 0.4; conf.low = -21; conf.high = 7; method = Wilcoxon rank sum test with continuity correction; alternative = two.sided	0.396	0.643

Impairment	Group	Feature	Method	Test output	p-value	p-value adjusted
		EBVEBNA IgG	KW/MW Test	estimate = -2; statistic = 740; p.value = 0.46; conf.low = -12; conf.high = 5; method = Wilcoxon rank sum test with continuity correction; alternative = two.sided	0.46	0.643
		HBSAGII	KW/MW Test	estimate = 3; statistic = 400; p.value = 0.66; conf.low = -10; conf.high = 16; method = Wilcoxon rank sum test with continuity correction; alternative = two.sided	0.658	0.658
		HSV-1	KW/MW Test	estimate = -3; statistic = 1700; p.value = 0.28; conf.low = -9; conf.high = 2; method = Wilcoxon rank sum test with continuity correction; alternative = two.sided	0.284	0.643
		HSV-2	KW/MW Test	estimate = -13; statistic = 310; p.value = 0.03; conf.low = -23; conf.high = -1.7e-05; method = Wilcoxon rank sum test with continuity correction; alternative = two.sided	0.0302	0.106
	Vitamins	B12 II	KW/MW Test	statistic = 3.2; p.value = 0.2; parameter = 2; method = Kruskal-Wallis rank sum test	0.2	0.481
		FOL III	KW/MW Test	statistic = 0.043; p.value = 0.98; parameter = 2; method = Kruskal-Wallis rank sum test	0.979	0.979
		VitD II	KW/MW Test	statistic = 2.3; p.value = 0.32; parameter = 2; method = Kruskal-Wallis rank sum test	0.321	0.481
